# METTL17 coordinates ferroptosis and tumorigenesis by regulating mitochondrial translation in colorectal cancer

**DOI:** 10.1016/j.redox.2024.103087

**Published:** 2024-02-13

**Authors:** Hao Li, Kailun Yu, Huilong Hu, Xiandan Zhang, Siyu Zeng, Jiawen Li, Xiaoning Dong, Xusheng Deng, Jianhui Zhang, Yongyou Zhang

**Affiliations:** a State Key Laboratory of Cellular Stress Biology, Innovation Center for Cell Signaling Network, Engineering Research Centre of Molecular Diagnostics of the Ministry of Education, School of Life Sciences, Xiamen University, Xiamen, Fujian, 361102, China; b National Institute for Data Science in Health and Medicine Engineering, Faculty of Medicine and Life Sciences, Xiamen University, Xiamen, Fujian, 361102, China; c School of Pharmaceutical Sciences, Xiamen University, Xiamen, Fujian 361102, China

**Keywords:** METTL17, Ferroptosis, Mitochondrial RNA methylation, Colorectal cancer (CRC)

## Abstract

Ferroptosis, an iron-dependent lipid peroxidation-induced form of regulated cell death, shows great promise as a cancer therapy strategy. Despite the critical role of mitochondria in ferroptosis regulation, the underlying mechanisms remain elusive. This study reveals that the mitochondrial protein METTL17 governs mitochondrial function in colorectal cancer (CRC) cells through epigenetic modulation. Bioinformatic analysis establishes that METTL17 expression positively correlates with ferroptosis resistance in cancer cells and is up-regulated in CRC. Depletion of METTL17 sensitizes CRC cells to ferroptosis, impairs cell proliferation, migration, invasion, xenograft tumor growth, and AOM/DSS-induced CRC tumorigenesis. Furthermore, suppression of METTL17 disrupts mitochondrial function, energy metabolism, and enhances intracellular and mitochondrial lipid peroxidation and ROS levels during ferroptotic stress. Mechanistically, METTL17 inhibition significantly reduces mitochondrial RNA methylation, including m^4^C, m^5^C, m^3^C, m^7^G, and m^6^A, leading to impaired translation of mitochondrial protein-coding genes. Additionally, the interacting proteins associated with METTL17 are essential for mitochondrial gene expression, and their knockdown sensitizes CRC cells to ferroptosis and inhibits cell proliferation. Notably, combined targeting of METTL17 and ferroptosis in a therapeutic approach effectively suppresses CRC xenograft growth in vivo. This study uncovers the METTL17-mediated defense mechanism for cell survival and ferroptosis in mitochondria, highlighting METTL17 as a potential therapeutic target for CRC.

## 1 Introduction

Ferroptosis is a regulated cell death caused by iron-dependent lipid peroxidation on cellular membranes, which is morphologically and mechanistically distinct from autophagy, apoptosis, and necrosis [1]. Increasing evidence supports the role of ferroptosis in regulating various human diseases, particularly cancer [2,3]. Compared to normal cells, cancer cells have a greater demand for iron to support their growth [4], which makes them potentially more susceptible to ferroptosis. Therefore, ferroptosis-based therapy, especially in combination with classic therapy, holds promise for cancers that are resistant to conventional therapies [[2], [3], [4], [5], [6]]. While numerous cancer-related signal pathways and genes influencing ferroptosis in cancer cells have been identified, and certain ferroptosis inducers have shown potential for cancer treatment, the precise regulation of ferroptosis in specific cancer types remains largely unknown.

Recent studies underscore the pivotal roles of mitochondria in ferroptosis regulation [[7], [8], [9], [10]]. Morphologically, ferroptotic cells are characterized by shrunken mitochondria and reduced numbers of mitochondrial cristae, accompanied by accumulation of mitochondrial lipid peroxidation and ROS [1]. Beyond the established prerequisites of ferroptosis, involving the synthesis and peroxidation of polyunsaturated fatty acid-containing phospholipids (PUFA-PL) and iron metabolism, mitochondrial metabolism contributes significantly to this process [3]. Upon cysteine deprivation, cells undergo hyperpolarization of mitochondrial membrane potential, lipid peroxide accumulation, and ferroptosis, with mitigation achieved by suppressing the mitochondrial tricarboxylic acid (TCA) cycle or electron transfer chain (ETC) [8]. Most cellular ROS originates from mitochondria, where superoxide generated by electron leakage from ETC complexes I and III are converted to H_2_O_2_ by superoxide dismutase [11]. The subsequent interaction of labile iron and H_2_O_2_ can interact through the Fenton reaction produces hydroxyl radicals, triggering PUFA-PL peroxidation [11,12]. Moreover, ATP synthesis, facilitated by proton pumping and electron transport within mitochondria, promotes ferroptosis [13,14]. Excessive accumulation of lipid peroxides can be naturally detoxified by cellular ferroptosis defense systems, which contain four known pathways, including the glutathione peroxidase 4 (GPX4)-reduced glutathione (GSH) system [15,16], the ferroptosis suppressor protein 1 (FSP1)-ubiquinol (CoQH2) system [17,18], the GTP cyclohydrolase1 (GCH1)-tetrahydrobiopterin (BH4) system [19,20], and the recently identified dihydroorotate dehydrogenase (DHODH)-CoQH2 system [9]. In mitochondria, DHODH cooperates with mitochondrial GPX4 to neutralize lipid peroxides [9]. However, whether there exist additional mitochondrial defense mechanisms against ferroptosis remains unclear. Ferroptosis resistance is observed in mitochondrial DNA-depleted ρ0 cells with mitochondrial translation defects, attributed to elevated expression of antioxidant enzymes, particularly the upregulation of mitochondrial GPX4 expression [10], suggesting the potential involvement of mitochondrial gene expression control in the regulation of ferroptosis.

Human mitochondria contain 1136 proteins (MitoCarta3.0) [21]. Most of them are encoded by the nuclear genome, translated in cytosol, and subsequently imported into mitochondria, while only 13 proteins are encoded by the mitochondrial genome and translated within mitochondria [21]. The mitochondrial genome consists of a 16.6 kb circular DNA (mt-DNA), encoding 13 proteins crucial for the oxidative phosphorylation (OXPHOS) complexes, 22 mitochondrial transfer RNAs (mt-tRNA), and two mitochondrial ribosomal RNAs (12S mt-rRNA and 16S mt-rRNA). RNA modification is a conserved and widespread phenomenon in all RNA species, including the mitochondrial RNA. The 22 mt-tRNAs are modified at 137 positions by 18 types of RNA modifications [22], and five types of modified residues exist in 12S mt-rRNA and 4 in 16S mt-rRNA [23]. The function of these mitochondrial RNA modifications is to determine efficient and accurate protein translation [24,25]. Mitochondrial RNA methylation is introduced in highly conserved sites by corresponding modifying enzymes, and many of these modifications and methyltransferases have been associated with human diseases, including cancer [24,26]. NSUN3 mediated-m^5^C34 methylation in mt-tRNAs drives the translation of mitochondrial mRNA to power cancer metastasis [27]. Similarly, METTL8-mediated m^3^C32 modification of mt-tRNAs optimizes tRNA structure and mitochondrial translation [[28], [29], [30]], with METTL8 overexpression associated with aggressive pancreatic cancer cells [31]. In 12S mt-rRNA, five methylation sites and corresponding methyltransferases (m^5^U429 by TRMT2B [32], m^4^C840 by METTL15 [33,34], m^5^C842 by NSUN4 [35,36], and m^6^_2_A936/m^6^_2_A937 by TFB1M [37])have been identified. Five modifications exist in 16S mt-rRNA (m^7^A947, Gm1145, Um1369, Um1370, and Ψ1397) [38,39]. METTL17, a member of the methyltransferase-like (METTL) family, reportedly methylated 12S mt-rRNA in m^4^C840 and m^5^C842, is essential for mouse embryonic stem cells (mESCs) differentiation [40]. Nevertheless, whether METTL17 still has methyltransferase activity in other sites requires further investigation. Furthermore, while METTL17 has been implicated in coactivating with estrogen receptors and regulating breast tumorigenesis [41], its functions in other cancers remains largely unknown.

Here, we investigate the biochemical mechanisms and molecular functions of METTL17 in ferroptosis regulation and cell survival determination of colorectal cancer (CRC). Interestingly, METTL17 expression is up-regulated in CRC and positively correlates with the resistance of CRC cells to ferroptosis, while METTL17 knockdown inhibits the oncogenic activity and increases the sensitivity to ferroptosis in CRC cells. Moreover, METTL17 plays a crucial role in colorectal tumorigenesis. Mechanistic analysis unveils that METTL17 knockdown leads to mitochondrial dysfunction by compromising mitochondrial gene expression. Inconsistent with previous studies, we identify that METTL17 is a mitochondrial protein targeting m^4^C, m^5^C, m^3^C, m^7^G, and m^6^A modification of mt-RNA. Additionally, METTL17-interacting proteins influence the sensitivity to ferroptosis and mitochondrial gene expression. Ultimately, combined targeting of METTL17 and ferroptosis proves effective in inhibiting CRC xenograft growth in vivo, presenting a promising therapeutic approach for CRC.

## 2 Results

### 2.1 METTL17 expression positively correlates with the resistance of cancer cells to ferroptosis, and the knockdown of METTL17 sensitizes CRC cells to ferroptosis

**Fig. 1:**
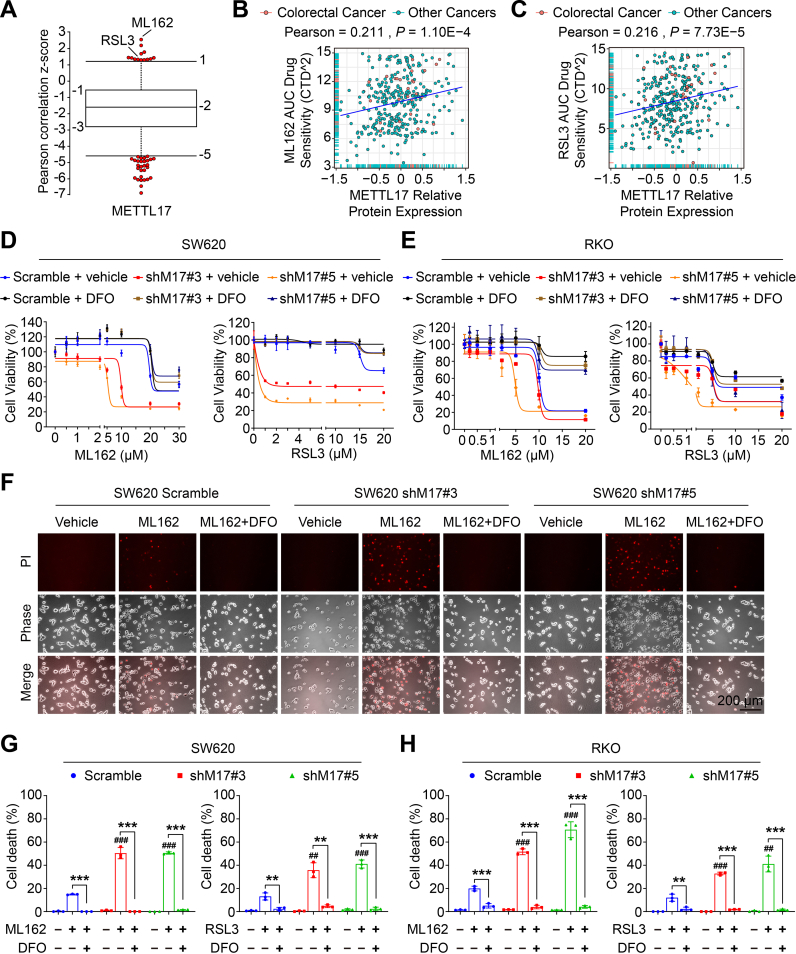
METTL17 expression is positively correlated with ferroptosis-resistance of cancer cells and knockdown of METTL17 sensitizes CRC cells to ferroptosis. **A.** METTL17 expression exhibited positive correlation with resistance to GPX4 inhibitors (ML162 and RSL3) in cancer cells. Data in plots were mined from the CTRP database and each plot represented a certain anti-tumor compound. **B and C.** Scatter plots showing the correlation between ML162 (B), RSL3 (C) AUC drug sensitivity and METTL17 protein level in colorectal cancer or other cancer cell lines. Data were mined from the DepMap database and each plot represented a certain cancer cell line. **D and E.** Cell viability of control (Scramble) and METTL17 knockdown (shM17#3 and shM17#5) SW620 cells (D) or RKO cells (E) treated with ML162 or RSL3 for 4 h after pretreatment with or without DFO (100 μM) for 1 h n = 3–4 per group. **F.** Microscopy showing cell death. SW620 cells were treated with ML162 (10 μM) for 4 h following pretreatment with or without DFO (100 μM) for 1 h. Upper panel: propidium iodide (PI) staining for dead cells; middle panel, phase-contrast; lower panel: merge image. Scale bar = 200 μm. **G and H**. Quantification of ferroptotic cells determined by PI-positive staining in SW620 cells (G) or RKO cells (H) treated with ML162 (10 μM) or RSL3 (10 μM) for 4 h following pretreatment with or without DFO (100 μM) for 1 h. The number of cell deaths was counted from Fig. 1F and Supplementary Fig. 1 n = 3 per group. Data are shown as mean ± SD. ^##^*p <* 0.01, ^###^*p <* 0.001 compared to ML162 or RSL3-treated Scramble group, and **p < 0.01, ***p < 0.001 compared with the indicated two groups, based on two-sided Student's *t*-test or Pearson *r* test.

The Cancer Therapeutics Response Portal (CTRP) provides a comprehensive dataset encompassing gene expression and drug resistance information for 907 cancer cell lines treated with 545 compounds [42]. Notably, analysis of CTRP data reveals a positive correlation between METTL17 mRNA expression and resistance to GPX4 inhibitors, including ML162 and RSL3, two classical inducers of ferroptosis (Fig. 1A). Further, comprehensive analysis of another two databases, Cancer Dependency Map (DepMap) [43] and Cancer Cell Line Encyclopedia (CCLE) [44], demonstrate that the protein level of METTL17 is positively associated to ML162 and RSL3 AUC drug sensitivity across various cancer cell lines (Fig. 1B and C).

**Fig. 2:**
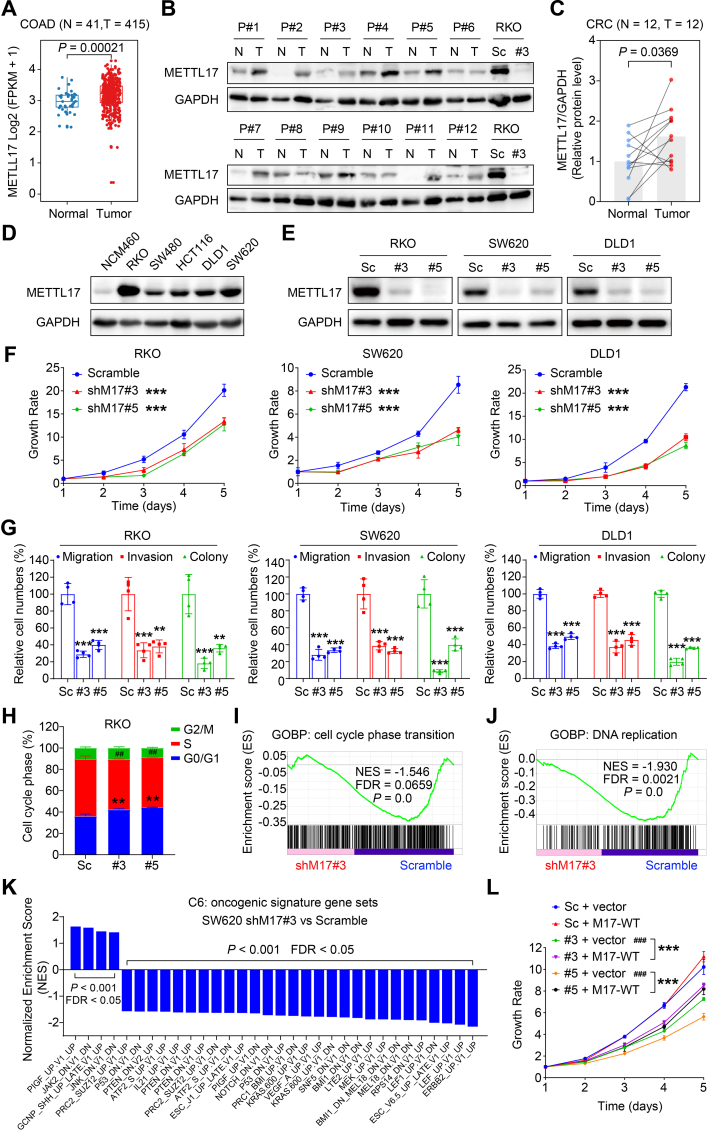
METTL17 is highly expressed in CRC and knockdown of METTL17 inhibits CRC cell proliferation, migration, invasion, and oncogenic signatures in vitro. **A.** The mRNA levels of METTL17 were up-regulated in colon adenocarcinoma (COAD) compared to adjacent normal tissues based on TCGA analysis. Normal tissue (N), n = 41. Tumor tissues (T), n = 415. **B and C.** The protein levels of METTL17 were measured in 12 pairs of human CRC tumor tissues (T) and the corresponding surrounding non-tumorous tissues (N) from 12 patients (abbreviated to P#1–12) (B). Relative protein level of METTL17 normalized to GAPDH was quantified by Image J (C). n = 12 per group. The METTL17 knockdown (#3) and Scramble (Sc) RKO cells were used as control. **D**. METTL17 was highly expressed in human CRC cell lines compared to human normal colonic epithelial cell line (NCM460) detected by western blotting. **E.** The knockdown efficiency of METTL17 in RKO, SW620, and DLD1 cells was confirmed by Western blot assays. Sc, Scramble. #3, shMETTL17#3. #5, shMETTL17#5. **F.** Crystal violet staining assays showed that knockdown of METTL17 reduced cell proliferation of RKO, SW620, and DLD1. n = 5–6 per group. shM17#3, shMETTL17#3. shM17#5, shMETTL17#5. **G.** Knockdown of METTL17 inhibited cell migration, invasion, and colony formation in RKO, SW620, and DLD1 cells measured through *trans*-well migration and invasion assays followed by crystal violet staining. The relative levels respectively were counted from Supplementary Fig. 3A, n = 4 per group. **H, I and J.** Knockdown of METTL17 caused cell cycle arrest at G0/G1 phase in RKO cells (H) (n = 3 per group), and GSEA analysis revealed disruptions in cell cycle phase transition (I) and DNA replication (J) in METTL17-deficient RKO cells. GOBP, Gene Ontology Biological Process. FDR, false discovery rate. **K.** GSEA analysis of RNA-Seq data identified that METTL17 knockdown in SW620 cells suppressed multiple oncogenic pathways. **L.** Exogenous overexpression of METTL17 via lentivirus rescued the inhibited cell proliferation in METTL17-deficient SW620 cells. n = 5–6 per group. M17-WT, human wide type METTL17. Data are shown as mean ± SD. ^##^*p <* 0.01, ^###^*p <* 0.001 compared to Scramble group, and **p < 0.01, ***p < 0.001 compared to Scramble group or the indicated two groups, based on two-sided Student's *t*-test or one-way ANOVA.

To determine whether METTL17 regulates ferroptosis in CRC, the lentivirus-mediated-shRNA knockdown of METTL17 was used to generate stable cell lines. Consistent with the observed correlations in the CTRP, DepMap, and CCLE datasets, knockdown of METTL17 in SW620 and RKO, two colorectal cancer cells expressing high levels of METTL17 (Fig. 2D), increased sensitivity to ML162 or RSL3-induced ferroptosis, while the cell viability was rescued by the iron chelator deferoxamine (DFO) (Fig. 1D and E). Additionally, propidium iodide (PI) staining revealed that ML162 or RSL3 treated METTL17-deficient cells exhibited more cell deaths, which were significantly reversed by DFO (Fig. 1F, G, H and Supplementary Fig. 1A, B, C), suggesting that knockdown of METTL17 sensitized CRC cells to GPX4 inhibition.

Next, we exogenously expressed METTL17 in METTL17-knockdown SW620 cells and found that restoring METTL17 expression partially rescued ML162-induced ferroptosis (Supplementary Fig. 2A, B, C). However, overexpression of METTL17 in Scramble cells slightly promoted ML162-induced ferroptosis (Supplementary Fig. 2A and B). In addition, METTL17 knockdown accelerated ferroptosis induced by Erastin, an inhibitor of the cystine/glutamate antiporter system Xc^−^ (Supplementary Fig. 2D), and restoring METTL17 expression partially rescued Erastin-induced ferroptosis in METTL17-knockdown RKO cells (Supplementary Fig. 2E and F). Collectively, these bioinformatic and experimental findings affirm the significant role of METTL17 in the regulation of ferroptosis in CRC.

### 2.2 METTL17 is overexpressed in human CRC and knockdown of METTL17 inhibits proliferation, migration, invasion and oncogenic signatures of CRC cells in vitro

The Cancer Genome Atlas (TCGA) analysis unveiled an up-regulation of METTL17 mRNA in colorectal cancer (CRC) tissues compared to normal colorectal tissues (Fig. 2A). Correspondingly, METTL17 protein expression was higher in CRC tissues than in their adjacent normal counterparts (Fig. 2B and C). Subsequently, the function of METTL17 in CRC was evaluated. Firstly, based on DepMap and CCLE databases, a varied distribution of METTL17 mRNA expression levels in all CRC cell lines was observed (Supplementary Fig. 12A), also indicating a correlation between METTL17 expression and its gene effect in CRC. Secondly, METTL17 protein levels were notably elevated in CRC cell lines compared to a normal colonic epithelial cell line (Fig. 2D). Among the CRC cell lines, RKO, SW620, and DLD1 exhibited relatively high METTL17 expression and were selected for subsequent phenotypic experiments.

To determine the role of METTL17 in CRC cell progression, we knocked down METTL17 expression in three human CRC cell lines SW620, RKO and DLD1 by using two different short hairpin RNAs against METTL17 and confirmed the knockdown efficiency via Western blot (Fig. 2E). The crystal violet assay demonstrated a significant inhibition of cell proliferation in METTL17-knockdown CRC cells (Fig. 2F). Additionally, cell migration and invasion assays revealed that down-regulation of METTL17 in RKO, SW620, and DLD1 cells dramatically suppressed their ability to migrate across membranes and invade through matrix-coated membranes (Fig. 2G and Supplementary Fig. 3A), indicating that METTL17 knockdown impedes the migration and invasion of CRC cells. Colony formation analysis further manifested that METTL17 knockdown significantly restrained CRC cell growth (Fig. 2G and Supplementary Fig. 3 A). Moreover, cell cycle analysis indicated that METTL17 knockdown led to prolonged G0/G1 stage and reduced S stage, suggesting that increased cell cycle arrest at the G0/G1 stage in METTL17-deficient CRC cells (Fig. 2H).

To elucidate the mechanisms by which METTL17 regulates CRC cell survival, we performed RNA-seq analysis on METTL17-knockdown and wild-type CRC cells, followed by Gene Set Enrichment Analysis (GSEA). In line with the observed cell cycle arrest at the G0/G1 stage in METTL17-deficient cells, GSEA revealed that METTL17-deficient CRC cells exhibited deficiencies in cell cycle phase transition (Fig. 2I) and DNA replication (Fig. 2J) compared to control cells. Moreover, we found that numerous oncogenic signature gene sets were down-regulated by METTL17 deficiency according to GSEA data (Fig. 2K). The impaired oncogenic signatures encompassed the ERBB2 pathway, LEF1 pathway, KRAS pathway, and MEK pathway (Supplementary Fig. 4A), in which the expression of oncogenic genes was down-regulated by METTL17 knockdown (Supplementary Fig. 4B). These findings collectively suggest that knockdown of METTL17 dramatically suppresses oncogenic activity in CRC cells.

Furthermore, we found that restoring METTL17 expression partially reversed the inhibited cell proliferation observed in METTL17-knockdown SW620 cells (Fig. 2L). Collectively, these results highlight that METTL17 is overexpressed in human CRC, and its knockdown inhibits proliferation, migration, invasion, colony formation and cell cycle transition of CRC cells, at least in part, through disrupting the oncogenic pathway.

### 2.3 METTL17 deficiency inhibits xenograft tumor growth and AOM/DSS-induced CRC tumorigenesis in vivo

**Fig. 3:**
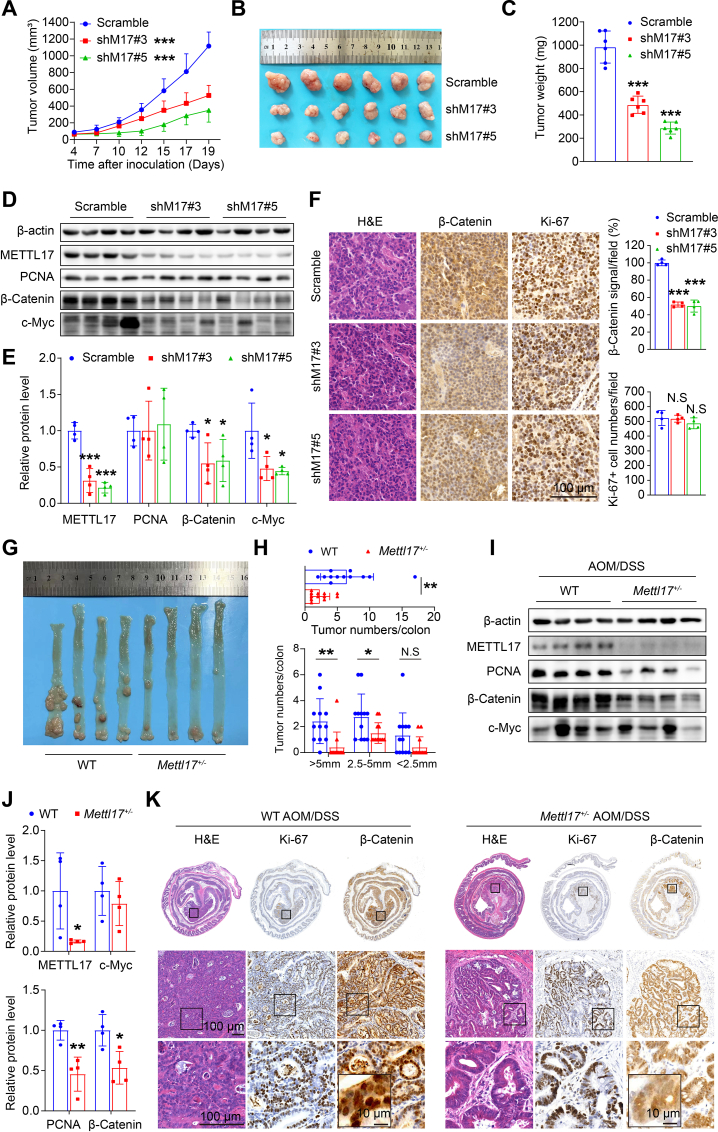
METTL17 deficiency reduces xenograft tumor growth and AOM/DSS-induced CRC tumorigenesis in vivo. **A, B and C.** Knockdown of METTL17 inhibited SW620 xenograft tumor growth (A) and reduced tumor size (B) and weights (C) in mice. n = 6 per group. **D and E.** Knockdown of METTL17 led to a reduction in the protein expression of β-Catenin and c-Myc, while the PCNA protein level remained unchanged, as analyzed by Western blot (D). The relative expression was quantified by normalizing to β-actin (E). n = 4 per group. **F.** Knockdown of METTL17 caused reduced β-Catenin expression, but unchanged Ki-67 positive cells in SW620 xenograft tumors. H&E, β-Catenin, and Ki-67 staining were performed on tissue sections from Scramble and METTL17 knockdown SW620 tumors (Scale bar = 100 μm). The β-Catenin signal and Ki-67-positive tumor cells were quantified in the 400 × field, n = 4 per group. **G and H.***Mettl17* heterozygote mice (*Mettl17*^+/−^) showed a decreased incidence of AOM/DSS-induced CRC formation compared to WT mice. Representative images of intact tumors were shown (G). *Mettl17*^+/−^ mice exhibited fewer and smaller colon tumors after AOM/DSS treatment, as indicated by the number of total tumors per colon (up) and different sizes' tumors per colon (down) (H). n = 12 per group. **I and J.** Colon tumors in *Mettl17*^+/−^ mice exhibited reduced levels of PCNA and β-Catenin proteins, with no significant change observed in c-Myc, as analyzed by Western blot (I). The relative expression was respectively quantified by normalizing to β-actin (J). n = 4 per group. **K.** H&E, Ki-67, and β-Catenin staining were performed on colon sections from WT and *Mettl17*^+/−^ mice after AOM/DSS treatment. Data are shown as mean ± SD. *p < 0.05, **p < 0.01, ***p < 0.001, based on two-sided Student's *t*-test.

To evaluate the impact of METTL17 in CRC tumorigenesis in vivo, we conducted a xenograft tumor model and an AOM/DSS-induced murine colitis-associated CRC model. Firstly, METTL17-knockdown and control SW620 cells were subcutaneously injected into nude mice. The results revealed that METTL17-knockdown tumors grew much more slowly and weighed much less than control tumors (Fig. 3A, B, C). Western blot and immunostaining analysis demonstrated that METTL17-knockdown tumors displayed reduced expression of β-Catenin, a marker of CRC progression, while the protein level of PCNA and the number of Ki-67 positive cells remained unchanged (Fig. 3D, E, F). These findings suggest that knockdown of METTL17 hinders tumor growth through proliferation-independent pathways in xenograft tumor model.

Secondly, we induced colitis-associated CRC through AOM/DSS treatment. Given the lethality observed in *Mettl17* knockout mice (Supplementary Fig. 5A, B, C), our experiments were performed with *Mettl17* heterozygotes (*Mettl17*^*+/−*^), which exhibited reduced METTL17 expression in the colonic epithelium without observable defects in colon development (Supplementary Fig. 5D and E). Interestingly, we showed that *Mettl17*^*+/−*^ protected mice from AOM/DSS-induced CRC tumorigenesis as evidenced by reduced tumor size and tumor number (Fig. 3G and H). Consistently, the protein expression levels of PCNA and β-Catenin were downregulated in *Mettl17*^*+/−*^ CRC samples (Fig. 3I and J). Next, we assessed the cell proliferation rate and malignancy grade through Ki-67 and β-Catenin immunostaining of colorectal tumors from both WT and *Mettl17*^*+/−*^ mice. The results revealed that METTL17 deficiency suppressed tumor progression, as *Mettl17*^*+/−*^ mice exhibited benign adenoma with well-differentiated glandular tubular structures and diffuse cytoplasmic distribution of β-Catenin, while WT mice displayed malignant tumors with hypo-differentiated carcinoma and nuclear localization of β-Catenin (Fig. 3K). These findings suggest that METTL17 deficiency inhibits xenograft tumor growth and AOM/DSS-induced CRC progression in vivo.

### 2.4 Knockdown of METTL17 disrupts mitochondrial homeostasis and promotes lipid peroxidation and ROS generation under ferroptotic stress

**Fig. 4:**
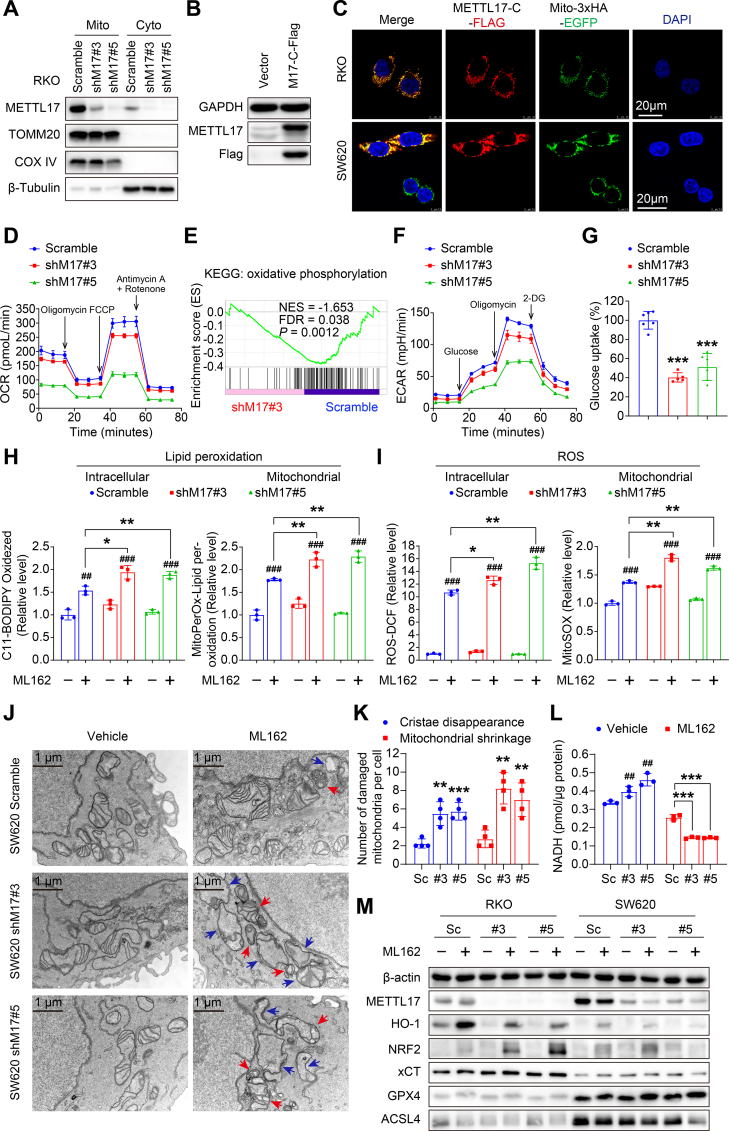
METTL17 loss causes significant mitochondrial dysfunction and promotes mitochondrial lipid peroxidation in ferroptosis. **A**. Western blot analysis revealed a higher expression of METTL17 in the mitochondrial fraction compared to the cytoplasm in RKO cells. Mito, mitochondria. Cyto, cytoplasm. **B.** Lentivirus mediated-overexpression of METTL17-C-Flag (M17-C-Flag) in RKO WT cells was confirmed by Western blot analysis. **C.** Immunofluorescence revealed co-localization between overexpressed METTL17 and mitochondria in RKO and SW620 cells, where mitochondria were stably tagged with 3xHA-EGFP (Scale bar = 20 μm). **D.** Seahorse analysis showed that METTL17 knockdown reduced oxygen consumption rate (OCR) in SW620 cells. n = 5–6 per group. **E.** GSEA analysis exhibited poor oxidative phosphorylation in METTL17 loss SW620 cells. The representative down-regulated genes were presented in Supplementary Fig. 13A. **F.** Seahorse analysis showed that METTL17 knockdown reduced extracellular acidification rate (ECAR) in SW620 cells. n = 5–6 per group. **G**. Knockdown of METTL17 significantly suppressed the glucose uptake ability of RKO cells. n = 5–6 per group. **H and I.** Knockdown of METTL17 increased intracellular and mitochondrial lipid peroxidation (H) and ROS (I) in ML162 (10 μM, 3 h)-treated SW620 cells, which were stained by indicated fluorescent dyes followed by flow cytometry detection. Relative levels were respectively quantified. n = 3 per group. **J and K.** Knockdown of METTL17 caused worse mitochondrial damage in ML162 (10 μM, 3 h)-treated SW620 cells detected by transmission electron microscope. Representative images were shown (Scale bar = 1 μm), with red arrows indicating mitochondrial shrinkage and blue arrows indicating the disappearance of mitochondrial cristae (J). The number of damaged mitochondria per cell was counted in ML162-treated SW620 cells (K), n = 4 per group. **L.** Knockdown of METTL17 in SW620 cells dramatically decreased cellular NADH content compared to Scramble cells after ML162 treatment (10 μM, 4 h), while METTL17 knockdown alone increased the level of NADH. n = 3 per group. **M.** Western blot analysis revealed alterations in ferroptosis markers mediated by METTL17 knockdown under ML162 (10 μM, 4 h) challenge in RKO and SW620 cells. The relative protein levels were performed semi-quantitative analysis, showing as a heatmap in Supplementary Fig. 6C. Data are shown as mean ± SD. ^##^*p <* 0.01, ^###^*p <* 0.001 compared to Scramble group, and *p < 0.05, **p < 0.01, ***p < 0.001 compared to Scramble group or the indicated two groups, based on two-sided Student's *t-*test.

To elucidate the mechanisms underlying the METTL17's impact on ferroptosis sensitivity and CRC development, we investigated the cellular distribution and function of METTL17 in cancer cells. Firstly, we separated RKO cells into mitochondrial and cytoplasmic fractions, conducting Western blot assays on both components to assess the endogenous METTL17 protein levels and cellular localization. Our findings indicated predominant expression of METTL17 in mitochondria (Fig. 4A), aligning with previous reports that METTL17, encoded by the nuclear genome, serves as a mitochondrial protein with an N-terminal transit peptide [45]. Secondly, since none of the available METTL17 antibodies met our immunofluorescence quality standards, we generated a C-terminal Flag-tagged METTL17 construct, which was overexpressed in CRC cells (Fig. 4B). Upon exogenous overexpression of METTL17-C-Flag in RKO and SW620 cell lines with stable expression of 3xHA-EGFP-tagged-mitochondria, we observed substantial METTL17-C-Flag localization in the mitochondria through fluorescence confocal microscopy (Fig. 4C). Thus, these results confirm the mitochondrial localization of METTL17.

To investigate the function of METTL17 in mitochondria, we examined alterations in energy metabolism in METTL17 knockdown cells. Initially, we measured the mitochondrial oxygen consumption rate (OCR) and observed a significant inhibition in OCR upon METTL17 knockdown, characterized by reductions in basal respiration, ATP production, maximal respiration, and spare capacity, particularly in shM17#5 cells (Fig. 4D). Consistently, GSEA analysis indicated a down-regulation of genes associated with oxidative phosphorylation in METTL17-deficient cells (Fig. 4E and Supplementary Fig. 13A), suggesting that METTL17 knockdown results to mitochondrial dysfunction. Subsequently, we assessed the extracellular acidification rate (ECAR) and identified diminished glycolytic capacity in METTL17 knockdown cells with reduced glycolytic reserve (Fig. 4F). Furthermore, we found that the knockdown of METTL17 significantly inhibited the glucose uptake ability of cancer cells (Fig. 4G), indicating impaired glucose supply in METTL17 loss cells. Collectively, these findings uncovered a critical role of METTL17 in maintaining mitochondrial homeostasis and glycolysis.

As the accumulation of ROS and oxidized lipids serve as a determinant of ferroptosis, mitochondrial events play crucial roles in this regulated cell death process [7,8,10,11]. Subsequently, we assessed cellular and mitochondrial levels of ROS and oxidized lipids in living cells using four fluorescent probes through flow cytometry and laser confocal microscopy, respectively. After 3 h of ML162 treatment, SW620 cells exhibited increased lipid peroxides, and METTL17 deficiency dramatically enhanced lipid peroxide formation under ferroptotic stress. The red fluorescence of the C11-BODIPY and MitoPerOx shifted to green fluorescence (Fig. 4H and Supplementary Fig. 6A and B), indicating excessive intracellular and mitochondrial lipid peroxidation in METTL17 knockdown cancer cells during ferroptotic stress. Concurrently, intracellular and mitochondrial ROS levels were significantly elevated in METTL17 knockdown cancer cells following ML162 treatment, as demonstrated by DCFH-DA and MitoSOX staining (Fig. 4I and Supplementary Fig. 6A and B).

Moreover, significant mitochondrial damage in METTL17 knockdown cells was found by transmission electron microscopy, characterized by increased mitochondrial shrinkage and reduced cristae after the ML162 challenge (Fig. 4J and K). We next investigated the mechanism of METTL17-regulated ferroptosis by detecting the protein levels of the key ferroptotic markers. Surprisingly, METTL17 knockdown dramatically suppressed heme oxygenase 1 (HMOX1/HO-1) expression and significantly upregulated NRF2 expression after ML162 treatment (Fig. 4M and Supplementary Fig. 6C). However, the key regulators of ferroptosis, including xCT, GPX4, and ACSL4, remained unchanged in METTL17-knockdown cells under both basal and ML162 treatment conditions (Fig. 4M). Additionally, we found that METTL17 knockdown alone was sufficient to increase NADH levels (Fig. 4L). However, under ferroptotic stress, METTL17 knockdown accelerated the decline of NADH (Fig. 4L), suggesting a disruption of the antioxidant system in METTL17-deficient cells during ferroptosis. In addition, METTL17 knockdown alone did not impact the expression of redox homeostasis-related genes (Supplementary Fig. 14A and B). Together, these results suggest that METTL17–mediated mitochondrial homeostasis is crucial for sustaining CRC growth and defending against ferroptosis.

### 2.5 METTL17 controls the mitochondrial translation of CRC in a mitochondrial RNA methylation manner

**Fig. 5:**
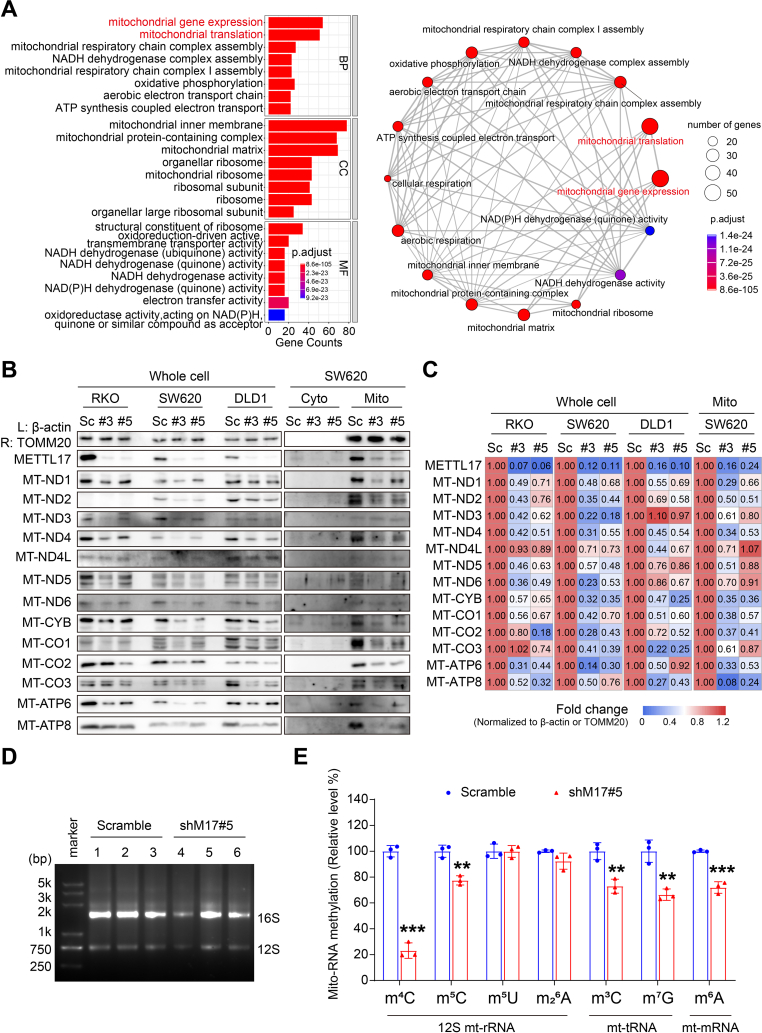
METTL17 is required for global mitochondrial translation in mitochondrial RNA methylation manner in CRC. **A.** GO enrichment analysis revealed that the top 100 METTL17 DepMap co-dependent genes predominantly associated with mitochondria, influencing the regulation of mitochondrial gene expression and mitochondrial translation. The bar plot illustrated enrichments in multiple mitochondria-related gene sets (left), while the visualized network highlighted the close connections among these gene sets (right). BP, biological process. CC, cellular component. MF, molecular function. **B and C.** Knockdown of METTL17 down-regulated the protein expression of mitochondrial coding genes in CRC cells detected by Western blot (B). Semi-quantitative analysis of the relative protein levels was performed and visualized in the heatmap of fold change, normalized to Scramble (C). **D and E.** The quality of mitochondrial RNA from RKO cells was identified by agarose gel assay (D). HPLC-MS analysis of mitochondrial RNA showed that knockdown of METTL17 significantly reduced m^4^C, m^5^C, m^3^C, m^7^G, and m^6^A levels within mitochondrial RNA methylations in RKO cells (E). Relative levels were quantified by comparing to scramble, n = 3 per group. Data are shown as mean ± SD. **p < 0.01, ***p < 0.001 compared to Scramble group, based on a two-sided Student's *t*-test.

METTL17 has been mentioned as a regulator of mitochondrial gene expression in mESCs [*40*]. However, it is unclear whether METTL17 is required for mitochondrial gene expression in cancer cells. Initially, we conducted a comprehensive analysis of METTL17's top 100 co-dependent genes from the DepMap database, which scores gene dependency across over 700 cancer cell lines through genome-wide pooled CRISPR-Cas9 knockout screens [43], and performed these genes to Gene Ontology (GO) analysis. The GO analysis revealed a notable enrichment of METTL17 co-dependent genes-encoding proteins located in the inner mitochondrial membrane and mitochondrial ribosome. These proteins play integral roles in coordinating mitochondrial gene expression and translation, impacting diverse processes such as mitoribosome biogenesis, NADH, ubiquinone, and quinone activity (Fig. 5A and Supplementary Fig. 15A). To validate the involvement of METTL17 in mitochondrial gene regulation, we directly examined the protein level of 13 mitochondrial protein-coding genes using the Western blot assays. The results demonstrated that METTL17 knockdown in CRC cells led to a global reduction in mitochondrial proteins in CRC cells (Fig. 5B and C), however, without a concurrent alteration in mRNA levels (Supplementary Fig. 7A). This indicates that METTL17 is necessary for maintaining regular mitochondrial gene expression in cancer cells.

While previous studies have indicated that METTL17 binds to 12S mt-rRNA and mediates m^4^C840 and m^5^C842 methylation in mESCs [40], its potential involvement in additional methyltransferase activities, particularly in mt-tRNAs and mt-mRNAs, has remained elusive. To address this gap in knowledge, we conducted LC-MS/MS analysis of mitochondrial RNA to explore alterations in methylation patterns in METTL17 knockdown CRC cells (Fig. 5D). Our findings revealed a significant reduction in m^4^C levels to approximately 20% in shM17#5 compared to control cells, while m^5^C, another RNA modification linked to METTL17, decreased to around 75% in METTL17-knockdown cells (Fig. 5E). Intriguingly, m^3^C, a modification previously associated with METTL8 in mt-tRNA [[28], [29], [30], [31]], exhibited a decrease to approximately 70% in METTL17-knockdown cells (Fig. 5E), suggesting a potential role for METTL17 as a yet-unidentified methylation regulator for m^3^C in mt-tRNA. Additionally, the levels of m^7^G and m^6^A were also diminished with METTL17 knockdown (Fig. 5E), indicating that METTL17 may be a multifunctional regulator for mitochondrial RNA methylation. Importantly, the knockdown of METTL17 did not affect the expression of other mitochondrial methyltransferases (Supplementary Fig. 8A). Collectively, our results propose that METTL17 governs the mitochondrial gene expression by modulating the methylation of m^4^C and m^5^C in 12S mt-rRNA, m^3^C and m^7^G in mt-tRNA, as well as m^6^A in mt-mRNA in CRC cells.

### 2.6 METTL17 interacting proteins regulate mitochondrial gene expression and ferroptosis

**Fig. 6:**
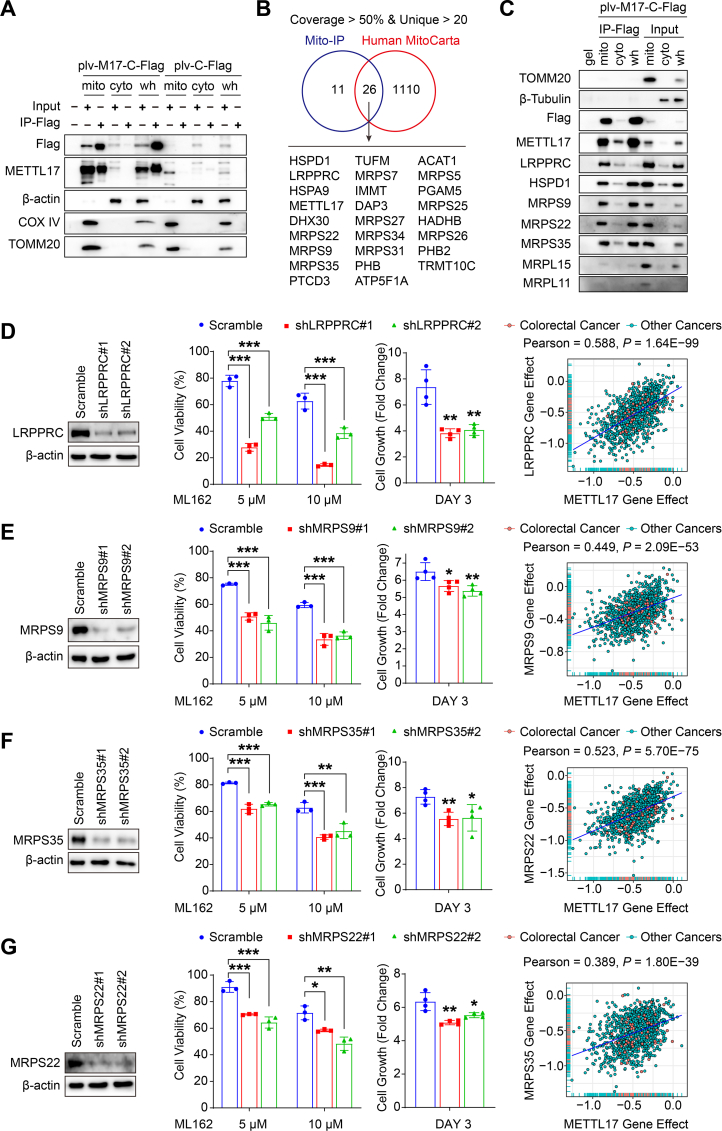
Targeting METTL17 interacting proteins sensitizes cancer cells to ferroptosis. **A.** RKO cells overexpressing METTL17-C-Flag through plv lentivirus (plv-M17-C-Flag) were isolated into mitochondrial (mito), cytoplasmic (cyto), and whole cell (wh) fractions. Immunoprecipitation with anti-Flag affinity gel (IP-Flag) was performed, and the samples were subsequently identified by Western blot. **B.** Mitochondrial precipitants (Mito-IP) were subjected to mass spectrometry analysis to identify the protein complexes binding to METTL17-C-Flag. Venn diagram analysis, based on mapping Mito-IP with Human MitoCarta, identified 26 METTL17 interactors with high affinity to METTL17, filtered by coverage >50% and unique peptide >20. **C.** RKO cells overexpressing METTL17-C-Flag (plv-M17-C-Flag) were isolated into mitochondrial (mito), cytoplasmic (cyto), and whole cell (wh) fractions, and immunoprecipitated by anti-Flag affinity gel (IP-Flag). Western blot analysis of METTL17 precipitants revealed strong interactions between METTL17 and LRPPRC, HSPD1, MRPS9, MRPS22, and MRPS35. **D, E, F and H.** Knockdown of METTL17-interacting proteins, LRPPRC (D), MRPS9 (E), MRPS35 (F), and MRPS22 (G) using lentivirus pLKO-shRNA, sensitized RKO cells to ML162-induced ferroptosis (n = 3 per group). Moreover, their knockdown inhibited cell proliferation in RKO cells (n = 4 per group), and the gene effect of METTL17 highly correlated with those of LRPPRC, MRPS9, MRPS35 and MRPS22. Data on gene knockout effect on cancer cells were mined from the DepMap database, with each plot representing a certain cancer cell line. Data are shown as mean ± SD. *p < 0.05, **p < 0.01 ***p < 0.001, based on two-sided Student's *t*-test or Pearson *r*-test.

Multiple proteins contribute to mitochondrial gene expression and protein translation. To uncover the interacting partners of METTL17, the mitochondrial, cytoplasmic and whole cell protein lysate were respectively enriched with Flag immunoprecipitation (IP) in RKO cells overexpressed METTL17-Flag by lentivirus (Fig. 6A and Supplementary Fig. 9A). The enriched proteins were subsequently identified through mass spectrometry (MS), with data filtered based on coverage greater than 50% and unique peptides exceeding twenty. A Venn diagram generated with Human MitoCarta, a mitochondrial proteome database comprising 1136 mitochondrial proteins in homo-sapiens [21], revealed 26 mitochondrial proteins exhibiting high-affinity interactions with METTL17 (Fig. 6B). GO enrichment analysis indicated that these proteins played essential roles in mitochondrial gene expression and mitochondrial translation (Supplementary Fig. 9B, C, D). Notably, IP–Western blot analysis demonstrated a robust affinity between METTL17 and MRPS9, MRPS22, and MRPS35, while a weaker interaction was observed with MRPL15 and MRPL11 (Fig. 6C). This suggests a specific interaction between METTL17 and the small subunit of the mitochondrial ribosome (MSSU), rather than the large subunit of the mitochondrial ribosome (MLSU). Besides MSSUs, our investigation identified a high interaction between METTL17 and LRPPRC (Fig. 6C), a mitochondrial protein essential for polyadenylation and coordination of translation in mitochondrial mRNAs [46].

To explore whether METTL17-interacting proteins also regulate ferroptosis and cell survival in CRC cells, we individually knocked down LRPPRC, MRPS9, MRPS22, and MRPS35 using lentiviral shRNA in RKO cells. Remarkably, similar to METTL17 knockdown experiments, the knockdown of these METTL17-interacting proteins sensitized cancer cells to ML162-induced ferroptosis and inhibited cell proliferation in CRC (Fig. 6D, E, F, G). Consistently, DepMap analysis revealed a strong correlation in gene effect between METTL17 and LRPPRC, MRPS9, MRPS22, and MRPS35, highlighting the significance of METTL17-associated cellular functions in cancer cell survival and ferroptosis defense (Fig. 6 D, E, F, G). Moreover, CTRP and DepMap analyses indicated that elevated expression of LRPPRC, MRPS9, MRPS22 and MRPS35 correlated with resistance to ML162 and RSL3 (Supplementary Fig. 10A-L). In addition, the knockdown of METTL17 did not affect the expression of these interacting proteins (Supplementary Fig. 9E and F). Notably, our findings unveiled a high correlation among METTL17-interacting proteins in governing cancer cell survival (Supplementary Fig. 11A).

Taken together, these results identify that METTL17 and its interacting proteins together regulate mitochondrial gene expression, which contributes to ferroptosis defense and cell survival in CRC cells.

### 2.7 Inhibition of CRC tumorigenesis by combination therapy targeting METTL17 and ferroptosis

**Fig. 7:**
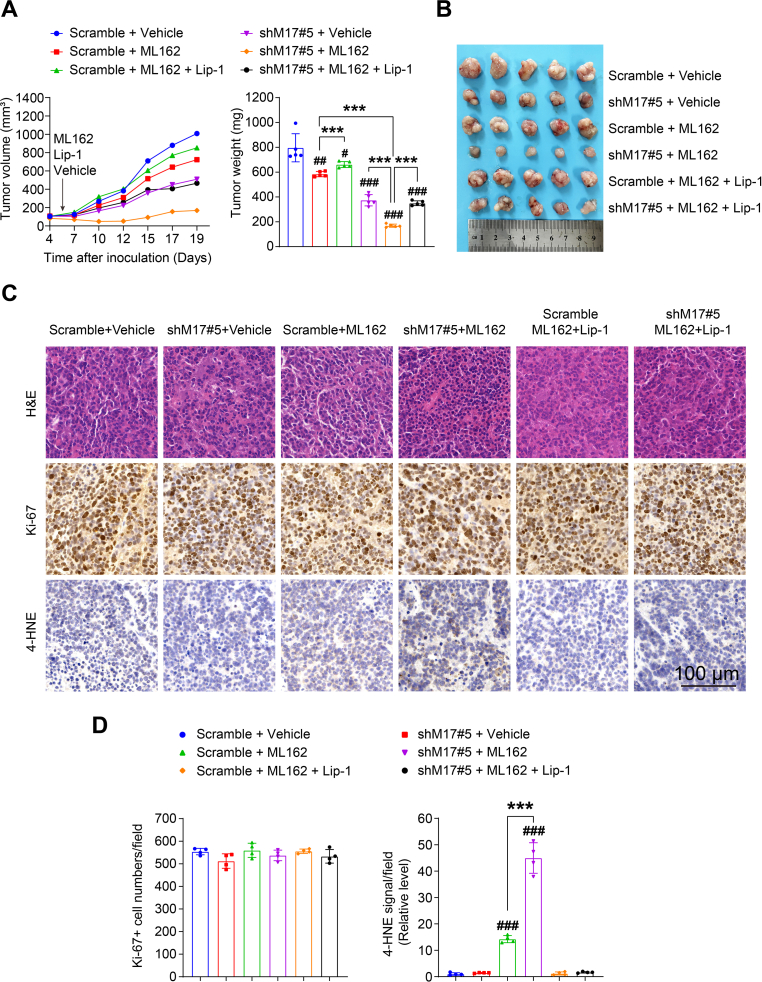
Combination therapy by targeting METTL17 and ferroptosis suppress CRC tumorigenesis. **A and B.** The tumor growth volume, weight, and size of SW620 xenograft tumors were significantly attenuated in the combination group receiving shRNA targeting METTL17 and ML162 treatment compared to monotherapy with either METTL17 knockdown or ML162 treatment. However, the efficacy of the combination treatment was compromised by the administration of Lip-1, a potent ferroptosis inhibitor by blocking lipid peroxidation. Vehicle control, ML162 (10 mg/kg per mouse) and Lip-1 (10 mg/kg per mouse) were intraperitoneally administrated every day starting from day 5 post SW620 inoculation. n = 5 per group. **C and D.** H&E, Ki-67, and 4-HNE staining of tissue sections from SW620 xenograft tumors (C). Scale bar = 100 μm. Ki-67-positive tumor cells and 4-HNE signal were quantified at the 400 × field (D), n = 4 per group. Data are shown as mean ± SD. ^#^*p <* 0.05, ^##^*p <* 0.01, ^###^*p <* 0.001 compared to Scramble vehicle group, and ***p < 0.001 compared to indicated two groups, based on two-sided Student's *t*-test.

Since METTL17 knockdown alone inhibited the growth of SW620 xenograft tumors in vivo and sensitized CRC cells to GPX4 inhibition in vitro, we further investigated the therapeutic potential of combined METTL17 inhibition and ferroptotic induction for CRC treatment. Initially, we examined whether METLL17 knockdown increases the sensitivity of CRC cells to a ferroptosis inducer. Indeed, METTL17 knockdown sensitized SW620 xenograft tumors to ML162 treatment, showcasing a synergistic effect for CRC therapy, as evidenced by the decrease in tumor volume, size and weight (Fig. 7A and B). Importantly, co-treatment with liproxstatin-1 (Lip-1), a well-established ferroptosis inhibitor via blockage of lipid peroxidation, diminished the growth inhibition of METTL17-knockdown tumors under ML162 treatment (Fig. 7A and B). In addition, ML162 treatment did not affect Ki-67 staining in these tumors, but significantly increased 4-HNE staining, a marker of lipid peroxidation, in METTL17-knockdown tumors compared to control tumors (Fig. 7C and D). In contrast, the enhancement of lipid peroxidation was diminished by Lip-1 administration in ML162-treated METTL17-knockout tumors (Fig. 7C and D). Collectively, these results demonstrate that simultaneous inhibition of METTL17 and ferroptosis inducer provides synergistic efficacy in CRC treatment via enhancing ferroptosis.

## 3 Discussion

**Fig. 8:**
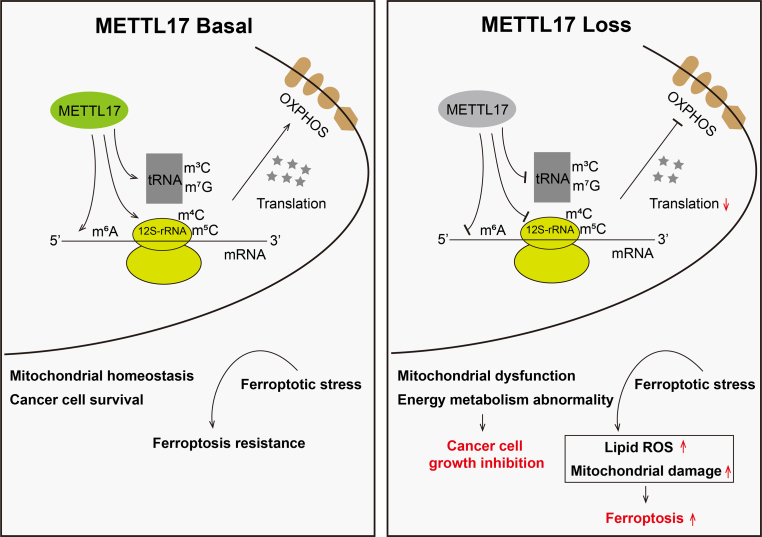
Schematic model for the mechanism by which METTL17 coordinates ferroptosis and tumorigenesis by regulating mitochondrial translation in CRC. METTL17 methylates m^4^C/m^5^C on 12S-rRNA, m^3^C/m^7^G on mt-RNA, and m^6^A on mt-mRNA within mitochondria, which affect mitochondrial translation efficiency and respiratory chain activity. METTL17 loss results in the suppression of these modifications and mitochondrial translation, leading to mitochondrial dysfunction and energy metabolism abnormality, thus inhibiting CRC cell growth and sensitizing CRC cells to ferroptosis.

Based on our studies, we can propose a model in which METTL17 coordinates ferroptosis and tumorigenesis by regulating mitochondrial translation in CRC (Fig. 8). As a methyltransferase, METTL17 methylates mitochondrial 12S rRNA in m^4^C and m^5^C, mitochondrial tRNA in m^3^C and m^7^G, as well as mitochondrial mRNA in m^6^A. Collaborating with interacting proteins, notably LRPPRC and MSSUs, METTL17 actively participates in the regulation of mitochondrial gene expression, there by upholding mitochondrial homeostasis. Genetic repression of METTL17 triggers dramatic mitochondrial dysfunction and energy metabolism abnormality, rendering CRC cells more susceptible to ferroptosis and impeding their growth. The concurrent targeting METTL17 and ferroptosis amplifies the efficacy of CRC suppression.

Recent studies have confirmed the pivotal role of mitochondria in the regulation of ferroptosis, with a majority of mitochondrial proteins identified as crucial regulators of this cellular process. Here, we demonstrate for the first time that high expression of mitochondrial METTL17 in tumor cells is positively correlated with resistance to ferroptosis inducers, which broadens the understanding of mitochondria-mediated ferroptosis. The methyltransferase-like (METTL) family is a varied group of methyltransferases, which were currently well characterized in cancer progression [47]. Also, we characterize for the first time that METTL17 is up-regulated in CRC and ablation of METTL17 significantly inhibits the oncogenic activity of CRC cells both in vitro and in vivo. Intriguingly, METTL17 knockdown exhibited negligible impact on proliferative markers PCNA and Ki-67 in xenograft, prompting speculation that METTL17 influences CRC progression through a proliferative-independent pathway, warranting further investigation. Notably, METTL17 knockdown effectively suppressed numerous oncogenic pathways in CRC, including KRAS signaling, ERBB2 signaling, LEF1 signaling, and MEK signaling. Additionally, our findings revealed that genetic ablation of METTL17 inhibited the protein level and nuclear location of β-Catenin in AOM/DSS-induced tumors, implying a crucial role of METTL17 in Wnt signaling during CRC development. We propose that the alteration of β-Catenin is due to the fact that deletion of METTL17 retards the development and progression of spontaneous CRC in mice. Moreover, we speculate that the METTL17-mediated regulation of global oncogenic activity might be the secondary effects due to its crucial role in maintaining mitochondrial homeostasis. However, the precise mechanisms through which METTL17 mediates various tumor pathways necessitate further exploration.

Multiple METTL family members are essential for fertilized egg formation, embryogenesis, and organ development [47]. METTL3, METTL14, or METTL1-deficient mice are embryonic lethality. Of note, in this study, we demonstrated that *Mettl17*^*−/−*^ mice were lethal during the embryonic stage, while *Mettl17*^*+/−*^ mice were procreative and developed normally with METTL17 knockdown in the colonic epithelium (Supplementary Fig. 5). Although our study confirmed that *Mettl17*^*+/−*^ protected mice from AOM/DSS-induced CRC, the possibility of specific systemic effects cannot be entirely dismissed. It will be interesting to further dissect out the role of METTL17 in CRC tumorigenesis in conditional knockout mice with tissue-specific METTL17 ablation in the future work.

Oxidative phosphorylation (OXPHOS) is the primary pathway for ATP generation in normal cells, while cancer cells predominantly rely on glycolysis for energy supply [48]. A genome-wide CRISPR death screen identified METTL17 as one of the candidates essential for OXPHOS and mitochondrial gene expression [49]. However, the definitive phenotype and underlying mechanisms remain elusive. Our mechanistic investigation unveiled that METTL17 knockdown in CRC cells led to significant mitochondrial dysfunction, coupled with a disruption in energy supply. This likely contributes to the inhibition of CRC tumorigenesis both in vitro and in vivo. Lipid peroxidation and ROS occur in both the cytosol and mitochondria during ferroptosis [1,3,9,11]. Upon ferroptotic stress, METTL17 knockdown cells exhibited exacerbated intracellular and mitochondrial lipid peroxidation and ROS formation. Additionally, examination of hallmark ferroptosis genes revealed down-regulation of HO-1 and up-regulation of NRF2 due to METTL17 knockdown under ferroptosis (Fig. 4M), indicating an imbalance in the antioxidant system mediated by METTL17 deficiency. Furthermore, the reducing equivalent NADH, crucial for the FSP1-CoQH2 ferroptosis defense system [17,18], was significantly reduced in METTL17 knockdown ferroptotic cells. In conclusion, the excessive lipid peroxidation and ROS formation occurring in both mitochondria and cytoplasm, along with disruptions in the antioxidant system should be the reasons why METTL17 deficiency sensitizes CRC cells to ferroptosis.

There was evidence that METTL17 deficiency in mESCs leads to mitochondrial ribosome assembly and protein translation defects, impacting MT-CO1 and MT-CO2 proteins, without affecting mitochondrial biogenesis or mt-DNA gene transcription [40]. Based on our bioinformatics analysis from the DepMap database, most of METTL17 co-dependent genes coordinate mitochondrial gene expression in cancer cells (Fig. 5A). In CRC cells, we observed that all of the 13 mitochondrial coding proteins were down-regulated by METTL17 knockdown. Interestingly, Mootha's lab showed that METTL17 binds to the mitoribosomal small subunit during late assembly and serves as an Fe–S cluster checkpoint, which promoting the mitochondrial translation [50]. Similarly in our research, we found that METTL17 strongly binds to mitochondrial ribosomal small subunit such as MRPS9, MRPS22 and MRPS35, which was also linked to mitochondrial translation. To our surprise, Mootha's lab firstly highlighted that METTL17 could bind to mitochondrial Fe–S clusters, which has recently been reported to regulate mitochondria-mediated ferroptosis by affecting lipid peroxidation [[51], [52], [53]]. In our findings, METTL17 loss caused much more serious mitochondrial lipid peroxidation during ferroptosis. Taken together, we hypothesize that the Fe–S cluster binding ability of METTL17 may connect mitochondrial translation to lipid peroxidation-mediated ferroptosis, the precise mechanisms however require further exploration.

A recent study revealed that targeting human mitochondrial RNA polymerase (POLRMT), crucial for OXPHOS system biogenesis, exhibited a potent anti-tumor response both in vitro and in vivo without affecting normal tissues [54]. This promising approach suggests that manipulating mitochondrial gene expression could be a viable strategy for cancer treatment, opening the door to METTL17 as a potential target for therapy. Additionally, our investigation into METTL17-interacting proteins shed light on their role in modulating sensitivity to ferroptosis and cell survival. Notably, LRPPRC, MRPS9, MRPS22, and MRPS35 exhibited not only a strong binding affinity with METTL17 but also a high co-dependency. Indeed, LRPPRC is overexpressed in various human tumors, which is associated with poor prognosis, and downregulation of LRPPRC suppresses oncogenic activity, triggers apoptosis, and overcomes drug resistance in cancer cells [55]. The MRPSs, essential for mitoribosome biogenesis, mitochondrial translation, and cellular respiration, are linked to tumor cell proliferation and cancer treatment [56]. The investigation of METTL17 and its interacting proteins provides new perspectives on targeting mitochondrial gene expression and ferroptosis in CRC treatment. However, the intricate relationship between mitochondrial gene expression defects and ferroptosis requires further investigation.

Recent studies have highlighted the importance of methylation and other modifications in highly conserved sites of mt-rRNA and mt-tRNA for mitochondrial ribosomal function and protein expression [24,27,31,57,58]. While several methyltransferases have been identified for mitochondrial RNA modifications, the full spectrum of their activities remains unclear. Although METTL17 was previously reported to specifically bind to 12S mt-rRNA and methylate the m^4^C840 and m^5^C842 sites in mESCs, its broader methyltransferase activity in other sites remained uncertain. In our investigation, employing LC-MS/MS analysis on mitochondrial total RNA, encompassing mt-rRNA, mt-tRNA, and mt-mRNA, we observed a substantial decline in m^4^C levels, a modest decrease in m^5^C, and slight reductions in m^3^C, m^7^G, and m^6^A levels following METTL17 knockdown in CRC cells. Notably, our study suggests a potential role for METTL17 in the regulation of m^3^C32 in mt-tRNA, a modification previously linked to mitochondrial METTL8 [[28], [29], [30], [31]]. However, rigorous biochemical assays are warranted for confirmation. While enzymes mediating m^7^G and m^6^A in mitochondria remain unidentified, our findings suggest that METTL17 can impact these modifications. Nevertheless, the precise coordination of these methylations by METTL17 should be further investigated. To assess mitochondrial RNA methylation, we utilized both in vitro cell model and in vivo tumor samples. However, despite our efforts to acquire high-quality mitochondrial RNA from tumor samples, we were unable to obtain sufficient quantities for methylation detection. This limited our ability to validate our in vitro findings with in vivo data, and further investigation using appropriate animal models is necessary to confirm the effects of METTL17 deficiency on mitochondrial RNA methylation in vivo. This study underscores METTL17's multifunctional role as a transmethylase in the regulation of mitochondrial RNA methylation, further research is needed to unravel the intricate mechanisms by which METTL17 governs various RNA modifications, both directly and indirectly.

Our study establishes the critical role of METTL17 as a mitochondrial RNA methyltransferase in CRC, coordinating ferroptosis and tumorigenesis through the regulation of mitochondrial translation. For the first time, we demonstrate that blocking METTL17 inhibits CRC tumorigenesis in vitro and in vivo and improves the efficiency of ferroptosis-based therapy in CRC treatment. Moreover, our findings reveal that targeting the METTL17-associated pan-mitochondrial translation regulation could expand our current concept of ferroptosis vulnerability in cancer therapy. Thus, the targeting METTL17, either alone or in combination with ferroptosis induction, holds particular promise for CRC therapy.

## 4 Methods and materials

### 4.1 Mice

Male nude BALB/c mice (6–8 weeks old) were used for xenograft tumor growth experiments. Male or female C57BL/6 *Mettl17*^*+/−*^ mice (6–8 weeks old) and littermate wild-type (WT) mice were used for AOM/DSS-induced CRC model. All animal protocols were approved by the Animal Care and Use Committee of Xiamen University.

### 4.2 Patient-derived colorectal tissue samples

Twelve pairs of human CRC specimens along with matched adjacent non-tumorous colorectal tissues were obtained from the Zhongshan Hospital of Xiamen University (Xiamen, China) for Western blot analyses. Prior to sample collection, informed consent was obtained from each patient.

### 4.3 Establishment of stable knockdown or overexpression cell lines

The knockdown of METTL17, LRPPRC, MRPS9, MRPS22 and MRPS35 in human CRC cell lines was performed though the use of short hairpin RNAs (shRNAs) cloned into pLKO.1-puro vector. The specific sequences are as follows: for METTL17, shMETTL17#3 (named shM17#3 or #3), 5′-ATGGCGTAAGACAGGTCATTT-3’. shMETTL17#5 (named shM17#5 or #5), 5′-GCATTTCAGCCACAAACTTTG-3’. shLRPPRC#1, 5′-GATGTGAGTCACTATAATGCT-3’. shLRPPRC#2, 5′-CCTCAAAGGAATGCAAGAATT-3’. shMRPS9#1, 5′-CCAGAAACTTTCACTCAAGAA-3’. shMRPS9#2, 5′-CCGTCCATTTCACTATCTCTT-3’. shMRPS22#1, 5′-GAAGGAACAGAAACAACCAAA-3’. shMRPS22#2, 5′-GCTACCTTGCTCTTTCGAGAT-3’. shMRPS35#1, 5′-GAGCACGAGTAGTAGTCTTAA-3’. shMRPS35#2, 5′-CGCTCCTTGGTACTAAAGAAAT-3’. Scramble, 5′-TCCTAAGGTTAAGTCGCCCTC-3’. The control shRNA was named as Scramble (or Sc). For overexpression of METTL17 in immunoprecipitation or immunofluorescence analyses, human METTL17 WT cDNA sequence was cloned into plv-C-Flag-puro vector, with METTL17 tagged Flag sequence in C-terminal. For overexpression of METTL17 into the METTL17-loss CRC cells, the human synonymous mutation of METTL17 cDNA sequence, according to the targets of shMETTL17#3 and shMETTL17#5, was cloned into plv-puro vector. The pLKO or plv clones, psPAX2 and pMD2.G packaging plasmids (4:3:1) were co-transfected into HEK293T cells for lentivirus packaging. After 48 h, lentivirus particles from the medium were collected and filtered. Subsequently, CRC cell lines were infected with lentivirus. 48 h later, infected cells were selected with puromycin to establish stable knockdown or overexpression cells.

### 4.4 Cell viability assay

Cell viability was assessed using the CellTiter-Glo Luminescent Cell Viability Assay (Promega #G9241). In brief, cells were seeded onto 96-well white plates at a density of 1 × 10^4^ per well for SW620 cells and 0.8 × 10^4^ per well for RKO cells. The next day, cells were treated with ML162, RSL3 or DFO at the indicated concentrations for 3–4 h. Subsequently, cell viability was determined according to the manufacturer's protocol.

### 4.5 Cell death assay

Cell death was measured by propidium iodide (PI) (SIGMA, Cat#537059) staining using a fluorescence microscope (ECHO RVL-100). For PI staining, cells were seeded at a density of 20–30% confluence into 12-well plates. Overnight, cells were treated with ML162, RSL3 or DFO at indicated concentrations for 3–4 h. To identify dead cells, PI (Final concentration: 500 μg/mL) was added to the medium for an additional 15 min. Subsequently, PI-positive dead cells were analyzed using a fluorescence microscope through TRITC fluorescent channel.

### 4.6 Cell proliferation assay

Cells were seeded at a density of 2 × 10^3^ per well in 24-well plates and harvested daily. The collected cells were fixed with 4% paraformaldehyde (PFA) for 15 min, stained with 0.1% crystal violet for 30 min, rinsed, and dried. After dissolving the stain in the cells for 30 min with 30% acetic acid, the solution was transferred to a transparent 96-well plate, and the absorbance at 540 nm was measured. The absorbance values recorded on the first day of growth served as the baseline, and fold changes were calculated using these values.

### 4.7 *Trans*-well migration and invasion assay

Migration and invasion experiments were performed using *trans*-well assays with Matrigel (invasion) or without Matrigel (migration). A total of 4 × 10^4^ cells were seeded into the top chambers in serum-free medium, while the 10%FBS medium was added to the bottom chambers. Following a 48-h incubation at 37 °C, the cells remaining on the top surface of the membrane were eliminated using cotton swabs. In contrast, the migrated cells on the bottom surface of the membrane were gently cleaned with PBS, fixed with methanol for 15 min, and stained with crystal violet for 30 min. After washing and air-drying, the migrated cells underneath the inserts were photographed using light microscopy at 200x magnification in five random fields. Three independent assays were used for analysis.

### 4.8 Antibodies and western blot analysis

Tumor tissues or cell samples were collected and homogenized in RIPA buffer (50 mM pH 8.0 Tris, 15 mM NaCl, 2 mM EDTA, 1% SDS, 0.5% sodium deoxycholate, 1% NP-40) supplemented with a protease inhibitor cocktail (APExBIO, Cat#K1007). Protein concentrations were measured using the BCA protein assay kit (TIANGEN, Cat#PA115-02). Approximately 30 μg of protein sample was separated on 8%, 10%, 12%, or 15% gel by SDS-PAGE electrophoresis and transferred to a PVDF membrane (Millipore, Cat#IPVH00010). The membranes were blocked with 5% skim milk in TBST (50 mmol/L Tris, 150 mmol/L NaCl, 0.5 mmol/L tris-buffered saline, and Tween-20, pH 7.5) for 1 h at room temperature. After blocking, the membranes were incubated with primary antibodies (1:1000 dilution, without indication) at 4 °C overnight and then washed with TBST, followed by incubation with secondary antibodies at room temperature for 1 h. Finally, the membranes were detected with ECL Western Blot Substrates (ThermoFisher SCIENTIFIC, Cat#34075) via C-Series Imaging System (Azure Biosystems C500). The signal intensity was quantified using the Image J software. The primary antibodies used for western blotting are as follows: Anti-METTL17 (BBI, Cat# D163865), Anti-β-Catenin (BD, Cat#610154), Anti-PCNA (Santa cruz, Cat# sc-56), Anti-c-Myc (Santa cruz, Cat#sc-40), Anti-β-actin (SIGMA, Cat#A5316), Anti-β-Tubulin (SAB, Cat#38075), Anti-TOMM20 (Abcam, Cat#ab186735), Anti-COX IV (ABclonal, Cat#A6564), Anti-GAPDH (SAB, Cat#37985), Anti-Flag (Abcam, Cat#ab205606), Anti-MT-ND1 (ABclonal, Cat#A17967), Anti-MT-ND2 (ABclonal, Cat#A17968), Anti-MT-ND3 (ABclonal, Cat#A17969), Anti-MT-ND4 (ABclonal, Cat#A9941), Anti-MT-ND4L (ABclonal, Cat#A17971), Anti-MT-ND5 (ABclonal, Cat#A12465), Anti-MT-ND6 (ABclonal, Cat#A17991), Anti-MT-CYB (ABclonal, Cat#A17966), Anti-MT-CO1 (ABclonal, Cat# A17889), Anti-MT-CO2 (ABclonal, Cat#A3843), Anti-MT-CO3 (ABclonal, Cat#A17891), Anti-MT-ATP6 (ABclonal, Cat#A8193), Anti-MT-ATP8 (ABclonal, Cat#A17890), Anti-LRPPRC (ABclonal, Cat#A3365), Anti-HSPD1 (ABclonal, Cat#A0564), Anti-MRPS9 (ABclonal, Cat#A11729), Anti-MRPS22 (ABclonal, Cat#A8319), Anti-MRPS35 (ABclonal, Cat#A15878), Anti-MRPL15 (ABclonal, Cat#A14400), Anti-MRPL11 (ABclonal, Cat#A4945), Anti-xCT/SLC7A11 (Abcam, Cat#ab175186), Anti-GPX4 (Abcam, Cat#ab125066), Anti-ACSL4 (Abcam, Cat#ab155282), Anti-NRF2 (Abcam, Cat#ab62352), Anti-HMOX1/HO-1 (ABclonal, Cat#A19062).

### 4.9 Subcutaneous xenograft model

A total of 5 × 10^6^ SW620 cells in 150 μL PBS was subcutaneously injected into the mouse dorsal flanks. One week after inoculation, the tumor size was measured along two perpendicular axes every other day using a vernier caliper. Three weeks after inoculation, tumors were isolated, weighted, and documented after euthanizing the mice. The formula (volume = length × width^2^ × 0.5) was used to calculate the volume of tumors. For the in vivo drug administration experiment, the mice were randomly divided into groups. ML162 (TOPSCIENCE, Cat#T8970) and Liproxstatin-1 (Lip-1) (TOPSCIENCE, Cat#T2376) were dissolved in vehicle (5% DMSO, 30% PEG300, 10% Tween-80 in saline). ML162 and Lip-1 were intraperitoneally injected at a dose of 10 mg/kg every day since the 4th day of tumor cell inoculation, and the vehicle was used as control. The mice were euthanized after two weeks of drug administration, and the subcutaneous tumors were isolated for further analysis.

### 4.10 AOM/DSS-induced mouse CRC model

Mice aged 6–8 weeks were received an intraperitoneal injection of azoxymethane (AOM, SIGMA, Cat#A5486) at the dose of 10 mg/kg. On the 7th day after AOM treatment, mice were given 2% dextran sodium sulfate (DSS, MP Biomedical, Cat#160110) dissolved in drinking water for 7 days, followed by a period of regular water for another 14 days. This cycle was repeated three times before all mice were sacrificed.

### 4.11 Lipid peroxide and ROS measurement

Intracellular ROS and lipid peroxide levels were measured using DCFH-DA (Beyotime, Cat#S0033 M) and C11-BODIPY 581/591 (Cayman Chemical, Cat#217075-36-0), respectively. Mitochondrial ROS and mitochondrial peroxidized lipid levels were measured using MitoSOX Red (Life Technologies, Cat#M36008) and MitoPerOx (Cayman Chemical, Cat#18798), respectively. Briefly, 2.5 × 10^5^ cells were seeded into each well of 12-well plates and cultured overnight. The next day, cells were treated with ML162 for 2–3 h, followed by adding probes into the culture medium for 30 min at 37 °C. After washing with PBS, cells were harvested by trypsinization, resuspended in 400 μL PBS, and analyzed using a flow cytometer (BD LSRFortessa). Data analysis was performed using FlowJo software. For fluorescence microscopy-based analysis, cells were seeded into a 35 mm confocal dish and cultured overnight. Following drug administration, cells were stained with probes for 30 min at 37 °C, and then images were documented using a laser scanning confocal microscope (Leica TCS SP8 DLS) to analyze intracellular and mitochondrial ROS and lipid peroxide levels in live cells.

### 4.12 Mitochondrial and cytosolic fractionation

Mitochondria and cytoplasm were isolated using the Cell Mitochondria Isolation Kit (Beyotime, Cat#C3601) according to the manufacturer's protocol. Briefly, cells were incubated in cold mitochondrial lyses buffer for 10 min, and the mixture was transferred into a glass homogenizer and homogenized for 40 strokes using a glass pestle on ice. The homogenate was centrifuged at 600 g for 10 min at 4 °C to remove unbroken cells and nuclei. Subsequently, the supernatant was collected and centrifuged at 12,000 g for 10 min at 4 °C to acquire the mitochondrial fraction (precipitate) and cytosolic fraction (supernatant). The mitochondrial fraction was dissolved in the buffer for further analysis.

### 4.13 Hematoxylin and eosin (H&E) staining, immunohistochemical analysis

Tissue samples from mice were immediately rinsed with cold PBS, fixed overnight with 4% PFA, and dehydrated in increasing gradients of ethanol and xylene. The specimens were then embedded in paraffin and sliced into 5 μm sections. For H&E staining, the tissue sections were dewaxed and stained with a standard H&E solution. For immunohistochemical staining, tissue sections were dewaxed, and antigen retrieval was performed in citrate buffer by heating in a boiling water bath (100 °C) in a microwave oven for 10 min. Endogenous peroxidase activity was blocked by incubating with 3% H_2_O_2_ for 10 min at room temperature. Subsequently, tissue sections were blocked with 5% goat serum for 30 min and incubated overnight at 4 °C with primary antibodies including anti-β-Catenin (BD, Cat#610154, 1:400 dilution), anti-Ki-67 (Abcam, Cat#ab279653, 1:1000 dilution), and anti-4-HNE (Abcam, Cat#ab46545, 1:300 dilution). After rinsing, the tissue sections were incubated with biotinylated goat anti-rabbit/mouse IgG for 10 min and with streptomycin-peroxidase for 10 min. Then, a DAB solution was added for colorization. Following a brief rinse, the sections were restrained briefly with hematoxylin for nuclear staining. Finally, the sections were dehydrated with ethanol and xylene, and sealed with neutral resin. Images were scanned at 200x or 400x magnification using a Motic VM1 microscope. The negative control group samples were treated according to the same protocol, except that the primary antibody was replaced with control IgG.

### 4.14 Immunofluorescence and confocal fluorescence microscopy

A total of 1 × 10^5^ RKO cells or SW620 cells were seeded in 24-well plates and allowed to grow overnight before treatment. The cells were fixed with 4% PFA for 15 min at room temperature and permeabilized on ice with 0.5% Triton X-100 in PBS buffer. The cells were blocked in PBS containing 5% BSA for 30 min at room temperature and then incubated with the appropriate primary antibody (1:200 dilution) at 4 °C overnight. Next, the cells were incubated with a FITC- or Texas Red-conjugated secondary antibody at room temperature for 1 h. Nuclei were stained with 4,6′-diamino-2-phenylindole (DAPI, SIGMA, Cat#D9542) for 5 min. Images were acquired with a 63x oil objective using a Leica SP8 (fluorescence) confocal microscope.

### 4.15 RNA sequencing (RNA-seq) analysis

The RNA of Scramble, shMETTL17#3, and shMETTL17#5 RKO or SW620 cells was extracted using an RNA extraction reagent kit (TIANGEN, Cat#DP419). Two independent samples (biological replicates) were established for each group. The RNA sequencing library was prepared and sequenced on a BGISEQ-500 platform (BGI, China) by SE50 sequencing. Sequencing reads were mapped to the human reference genome using the STAR aligner, and HTSeq calculated gene expression counts. Differential expression analysis was performed using the edgeR package, and the pathways enriched in the differentially regulated genes were analyzed by GSEA (http://software.broadinstitute.org/gsea/index.jsp). The sequencing data generated in this study are available at NCBI Gene Expression Omnibus (GEO, accession number GSE239325).

### 4.16 Mitochondrial RNA methylation detection

The mitochondria from Scramble and shMETTL17 RKO cells were isolated, followed by RNA extraction. A total of 400 ng mitochondrial RNA samples was digested with 1 unit of nuclease P1 (NEB, Cat#M0660S) in 30 μL NEB buffer at 37 °C for 2 h. To dephosphorylate the single nucleotides, 1 unit of rSAP (NEB, Cat#M0371S) was added and incubated at 37 °C for 2 h. The samples were then added with 200 μL nuclease-free water and centrifuged at 4 °C for 10 min. The supernatants were collected for loading into LC-MS/MS (AB SCIEX QTRAP6500+). Nucleosides were quantified using nucleoside-to-base ion mass transitions and retention time, referencing standards of m^4^C (TRC, Cat#B426568), m^5^C (TOPSCIENCE, Cat#T5912), m^3^C (TOPSCIENCE, Cat#T7351), m^6^_2_A (MCE, Cat#HY-101984), m^5^U (TRC, Cat#389366), m^7^G (TOPSCIENCE, Cat#T7319), and m^6^A (TOPSCIENCE, Cat#T6599).

### 4.17 OCR and ECAR measurements with Seahorse technology

A total of 2 × 10^4^ cells per well were seeded into an Agilent Seahorse 96-well XF Cell Culture Microplate. After 24 h of culture at 37 °C in 5% CO_2_ atmosphere, the culture medium was replaced with 175 μL of pre-warmed at 37 °C serum-free Seahorse XF Base Medium (pH 7.4; Agilent Technologies, Cat#102353-100) supplemented with 25 mM glucose, 0.5 mM pyruvate, and 4 mM glutamine for measurement of mitochondrial oxidative metabolism (OCR) or with 4 mM glutamine for measure of glycolysis (ECAR). The cells were then cultured in a CO_2_-free incubator for 1 h at 37 °C. The oxygen- and pH-sensitive probes were pre-incubated in a calibration solution (Agilent Technologies, Cat#100840-000) for 12 h at 37 °C in a CO_2_-free incubator. Oxygen consumption rate (OCR) and extracellular acidification rate (ECAR) were analyzed in a time course before and after injecting the following compounds successively. For OCR measurement, 1 μM Oligomycin, 0.5 μM FCCP, and 1 μM Antimycin A plus rotenone were used. For ECAR measurement, 12.5 mM Glucose, 1 μM Oligomycin, and 100 mM 2-deoxyglucose (2-DG) were required. A volume of 25 μL of the compound was injected into the well at a specific time. OCR and ECAR were then measured by Seahorse XFe96 analyzer, and the results were analyzed using Wave software.

### 4.18 Glucose uptake assays

The glucose uptake ability of CRC cells was determined using Glucose Uptake-Glo™ Assay (Promega, Cat#J1341) following the manufacturer's instructions.

### 4.19 Statistical analysis

All statistical analyses were performed using GraphPad Prism 8.0 (San Diego, CA, USA). Bioinformatics analysis was performed with R (version 4.2.1) (http://www.R-project.org/). The number of biological replicates for each experiment was indicated in the corresponding figure legend. Unless otherwise specified, all values represented the mean ± standard deviation (SD) and were derived from a minimum of three independent biological replicates. A two-tailed unpaired *t*-test was used for all analyses. In figures, statistical comparisons between the control group and the experimental group are denoted as follows: *p < 0.05, **p < 0.01, ***p < 0.001; ^#^*p <* 0.05, ^##^*p <* 0.01, ^###^*p <* 0.001, and N.S. (not statistically significant).

## CRediT authorship contribution statement

**Hao Li:** Writing – original draft, Supervision, Methodology, Investigation, Formal analysis, Data curation. **Kailun Yu:** Investigation, Data curation. **Huilong Hu:** Investigation. **Xiandan Zhang:** Investigation. **Siyu Zeng:** Investigation. **Jiawen Li:** Investigation. **Xiaoning Dong:** Investigation. **Xusheng Deng:** Software, Data curation. **Jianhui Zhang:** Investigation. **Yongyou Zhang:** Writing – review & editing, Supervision, Resources, Project administration, Funding acquisition, Formal analysis, Conceptualization.

## Declaration of competing interest

The authors declare no competing interests.

## Data Availability

Data will be made available on request.
