# Supplementary material for: METTL17 coordinates ferroptosis and tumorigenesis by regulating mitochondrial translation in colorectal cancer
Source: Redox Biol. 2024 Feb 13;71:103087. doi: 10.1016/j.redox.2024.103087 (PMC10884776; doi:10.1016/j.redox.2024.103087)

METTL17 coordinates ferroptosis and tumorigenesis by regulating mitochondrial translation in colorectal cancer

Hao Li[1], Kailun Yu[1], Huilong Hu[1], Xiandan Zhang[1], Siyu Zeng[1], Jiawen Li[1], Xiaoning Dong[1], Xusheng Deng[1], Jianhui Zhang[1], Yongyou Zhang[1,2#]

[1] State Key Laboratory of Cellular Stress Biology, Innovation Center for Cell Signaling Network, Engineering Research Centre of Molecular Diagnostics of the Ministry of Education, School of Life Sciences, Xiamen University, Xiamen, Fujian 361102, China

[2] National Institute for Data Science in Health and Medicine Engineering, Faculty of Medicine and Life Sciences, Xiamen University, Xiamen, Fujian 361102, China

[#] Address correspondence to authors at:
School of Life Sciences, Xiamen University, Xiamen, Fujian 361102, China. Fax: 86-0592-2187363; Email: yongyouzhang@xmu.edu.cn (Dr. Yongyou Zhang)

Supplementary Figure Legends

Supplementary Fig. 1. Knockdown of METTL17 sensitizes CRC cells to GPX4 inhibition.

A, B and C. Microscopy images showed cell death induced by ferroptotic stress. SW620 cells were treated with RSL3 (10 μM) for 4 h following pretreatment with or without DFO (100 μM) for 1 h (A). RKO cells were treated with ML162 (10 μM) or RSL3 (10 μM) for 4 h following pretreatment with or without DFO (100 μM) for 1 h (B, C). Upper panel: propidium iodide (PI) staining for dead cells; middle panel, phase-contrast; lower panel: merge image (Scale bar = 200 μm). The quantification of ferroptotic cells, determined by PI-positive staining, was presented as a percentage in Fig. 1 G and H.

Supplementary Fig. 2. Overexpression of METTL17 diminishes ferroptosis in METTL17 loss CRC cells.

A, B and C. Overexpression of METTL17 in METTL17-loss SW620 cells diminished ML162-induced ferroptosis. Cell viability of SW620 cells treated with ML162 (10 μM or 20 μM) for 4 h (A, B). n = 3 per group.Western blot for analyzing the expression of METTL17 in SW620 cells (C).

D. Knockdown of METTL17 sensitized RKO cells to Eratin-induced ferroptosis. Cell viability of RKO cells treated with the indicated dose of Erastin for 24 h. n = 3 per group.

E and F. Overexpression of METTL17 in METTL17-loss RKO cells diminished Erastin-induced ferroptosis. Cell viability of RKO cells treated Erastin (10 μM) for 24 h (E). n = 3 per group. Western blot for analyzing the expression of METTL17 in RKO cells (F).

Data are shown as mean ± SD. $^{\#}p < 0.05$, $^{\#\#}p < 0.01$, $^{\#\#\#}p < 0.001$ compared to Scramble group, and $^{*}p < 0.05$, $^{**}p < 0.01$, $^{***}p < 0.001$ compared with indicated two groups or the corresponding dose of Erastin-treated group, based on two-sided Student's *t*-test.

Supplementary Fig. 3. Knockdown of METTL17 inhibits migration, invasion, and colony formation of CRC cells.

A. Knockdown of METTL17 inhibited cell migration, invasion and colony formation of RKO, SW620, and DLD1 cells, as demonstrated by trans-well migration and invasion assays, followed by crystal violet staining (Scale bar = 200 μm). The relative levels were quantified for Fig. 2G, n = 4 per group.

Supplementary Fig. 4. Knockdown of METTL17 inhibits oncogenic pathways.

A and B. GSEA analysis of RNA-Seq data revealed that knockdown of METTL17 in SW620 cells suppressed multiple oncogenic pathways, including signatures such as ERBB2_UP.V1_UP, LEF1_UP.V1_UP, MEK_UP.V1_UP, and KRAS.600_UP.V1_DN (A). The heatmap displayed representative significant genes down-regulated by METTL17 knockdown (B). #3 or shM17#3 refers to shMETTL17#3.

Supplementary Fig. 5. *Mettl17*$^{-/-}$ mice are nonviable, while *Mettl17*$^{+/-}$ mice exhibit normal colon development.

A. Schematic diagram illustrating the generation of *Mettl17*$^{+/-}$ mice.

B. Genotyping analysis for the identification of *Mettl17*$^{+/-}$ mice.

C. Distribution graph representing the offspring after mating *Mettl17*$^{+/-}$ mice.

D. Western blot showing METTL17 expression in colonic epithelial cells of WT and *Mettl17*$^{+/-}$ mice.

E. H&E staining of colon sections obtained from WT and *Mettl17*$^{+/-}$ mice. Scale bar = 100 μm.

Supplementary Fig. 6. METTL17 knockdown promotes lipid peroxidation and ROS formation in both the cytoplasm and mitochondria.

A and B. Knockdown of METTL17 increased intracellular and mitochondrial lipid peroxidation and ROS levels in ML162 (10 μM, 3 h)-treated SW620 cells detected

by flow cytometry-(A) or in ML162 (10 μM, 3 h)-treated RKO cells detected by laser scanning confocal microscope (B). Scale bar = 25 μm. The CRC cells were stained by indicated fluorescent dyes.

C. The heatmap of fold change showed the semi-quantitative analysis of protein expression levels measured from the western blotting data presented in Fig. 4M.

Supplementary Fig. 7. Knockdown of METTL17 doesn't affect RNA levels of mitochondrial mRNA.

A. RT-qPCR analysis of mitochondrial mRNA in RKO cells. n = 3 per group.

Data are shown as mean ± SD. *$p < 0.05$, based on a two-sided Student's *t*-test.

Supplementary Fig. 8. Knockdown of METTL17 doesn't affect the expression of mitochondrial RNA methyltransferase.

A. Heatmap from RNA-Seq data illustrated that the expression of mitochondrial RNA methyltransferase remains unaffected by the knockdown of METTL17 in CRC cells.

Supplementary Fig. 9. METTL17 interacting proteins are essential for mitochondrial gene expression and knockdown of METTL17 doesn't affect their expression in CRC cell lines.

A. The fractions of mitochondria (mito), cytoplasm (cyto), and whole cell (wh) in RKO cells overexpressing METTL17-C-Flag (plv-M17-C-Flag), were isolated, immunoprecipitated using anti-Flag affinity gel, and subsequently identified by silver staining.

B, C and D. GO enrichment analysis showed that the 26 METTL17 interacting proteins regulated mitochondrial gene expression and translation. The bar plot illustrated enrichments in multiple mitochondria-related gene sets (B), while the visualized network highlighted the close connections among these gene sets (C). The gene-concept network depicted the linkages of METTL17-interacting proteins

and biological concepts (D). BP, biological process. CC, cellular component. MF, molecular function.

E and F. METTL17 knockdown did not affect the expression of METTL17 interacting proteins, as assessed by Western blot (E) and confirmed by heatmap analysis of RNA-Seq data (F).

Supplementary Fig. 10. The expression of LRPPRC, MRPS9, MRPS22, and MRPS35 positively correspond with cancer cell resistance to ferroptosis.

A, D, G and J. High expression of LRPPRC (A), MRPS9 (D), MRPS22 (G), and MRPS35 (J) correlated with resistance to GPX4 inhibitors (ML162 and RSL3) in cancer cells. Plotted data were mined from the CTRP database and each plot represented a certain anti-tumor compound.

B, C, E, F, H, I, K and L. Scatter plots showing the correlation between ML162, RSL3 AUC drug sensitivity and protein levels of LRPPRC, MRPS9, MRPS22, and MRPS35 in colorectal cancer or other cancer cell lines. Data were mined from the DepMap database and each plot represented a certain cancer cell line.

Correlation analysis was performed using Pearson *r* test.

Supplementary Fig. 11. DepMap analysis shows a high correlation between METTL17 and its interacting proteins in dependency gene effects of cancer cells.

A. Correlation plots demonstrated a robust association between METTL17-interacting proteins in regulating cell survival. Dot plots and Pearson coefficients along the diagonal represented the relationship between the two genes. Correlation analysis was performed using Pearson *r* test.

Supplementary Fig. 12. The relationship between the expression and gene effect of METTL17 in CRC cell lines.

A. Scatter plot illustrating the relationship between the expression and gene effect of METTL17 in CRC cell lines, mined from DepMap and CCLE database. Each dot

represented one certain colorectal cancer cell line, n = 55. A varied distribution of METTL17 mRNA expression levels in all CRC cell lines was observed.

Supplementary Fig. 13. METTL17 loss inhibits the gene expression related to oxidative phosphorylation.

A. The representative significant genes down-regulated by METTL17 knockdown in SW620 cells were shown by heatmap, corresponding to the GSEA analysis in Fig.4E.

Supplementary Fig. 14. The expression profiling of genes related to cell redox homeostasis.

A and B. Heatmaps illustrating the expression profiles of genes associated with cell redox homeostasis in METTL17 knockdown RKO cells (A) and SW620 cells (B). The analysis was based on the GSEA gene set labeled "GOBP cell redox homeostasis," encompassing 41 genes.

Supplementary Fig. 15. The METTL17 co-dependent genes enrich in the mitochondrial related pathways.

A. The co-dependent genes of METTL17 were enriched in the pathways of mitochondrial gene expression, mitochondrial translation, mitochondrial respiratory chain complex assembly, and NADH dehydrogenase complex assembly, as revealed by the gene-concept network analysis. The gene-concept network illustrated the connections between METTL17 co-dependent genes and biological concepts.

Supplementary Fig.1 Knockdown of METTL17 sensitizes CRC cells to GPX4 inhibition.

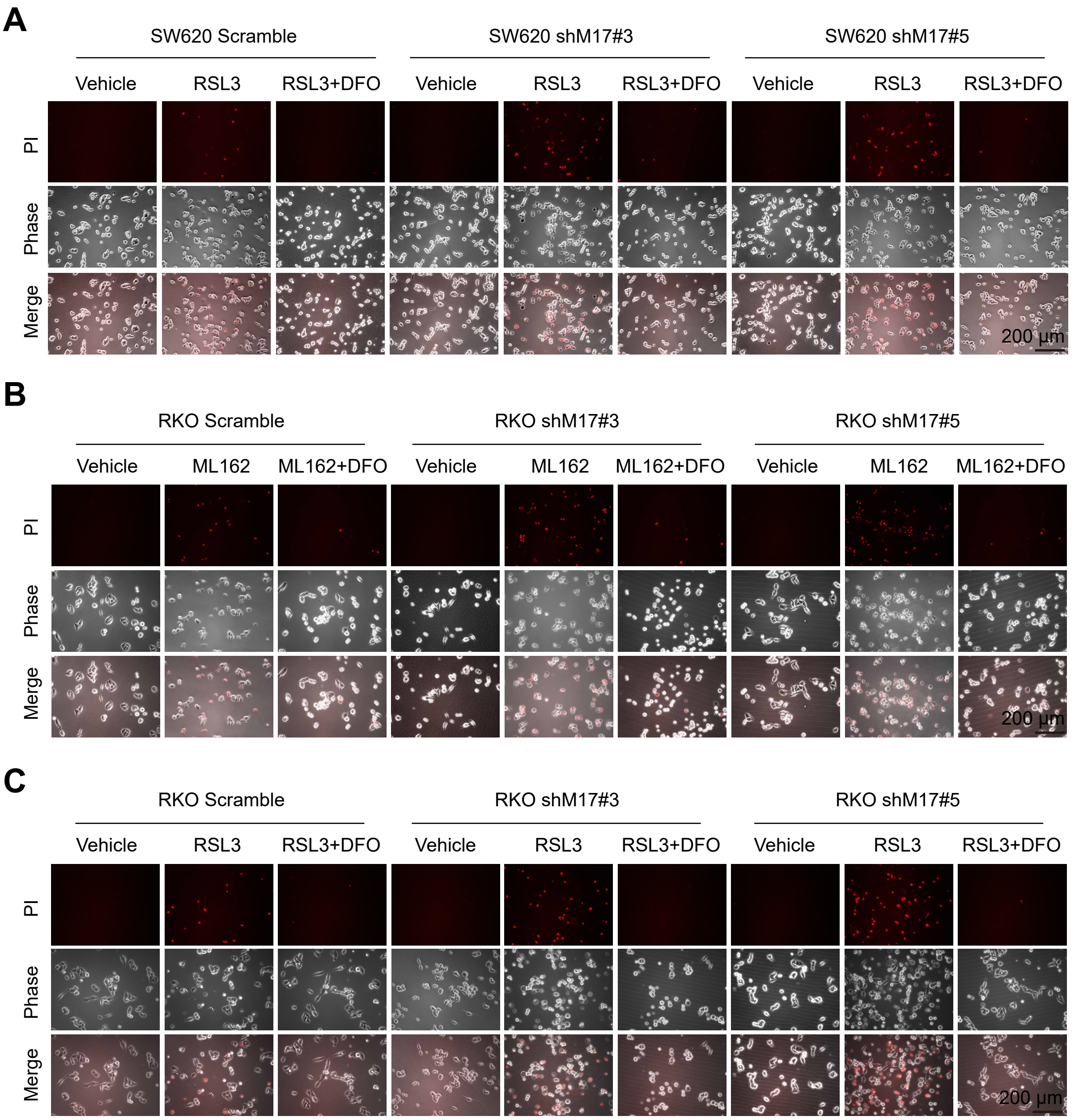

Supplementary Fig.2 Overexpression of METTL17 diminishes ferroptosis in METTL17 loss CRC cells.

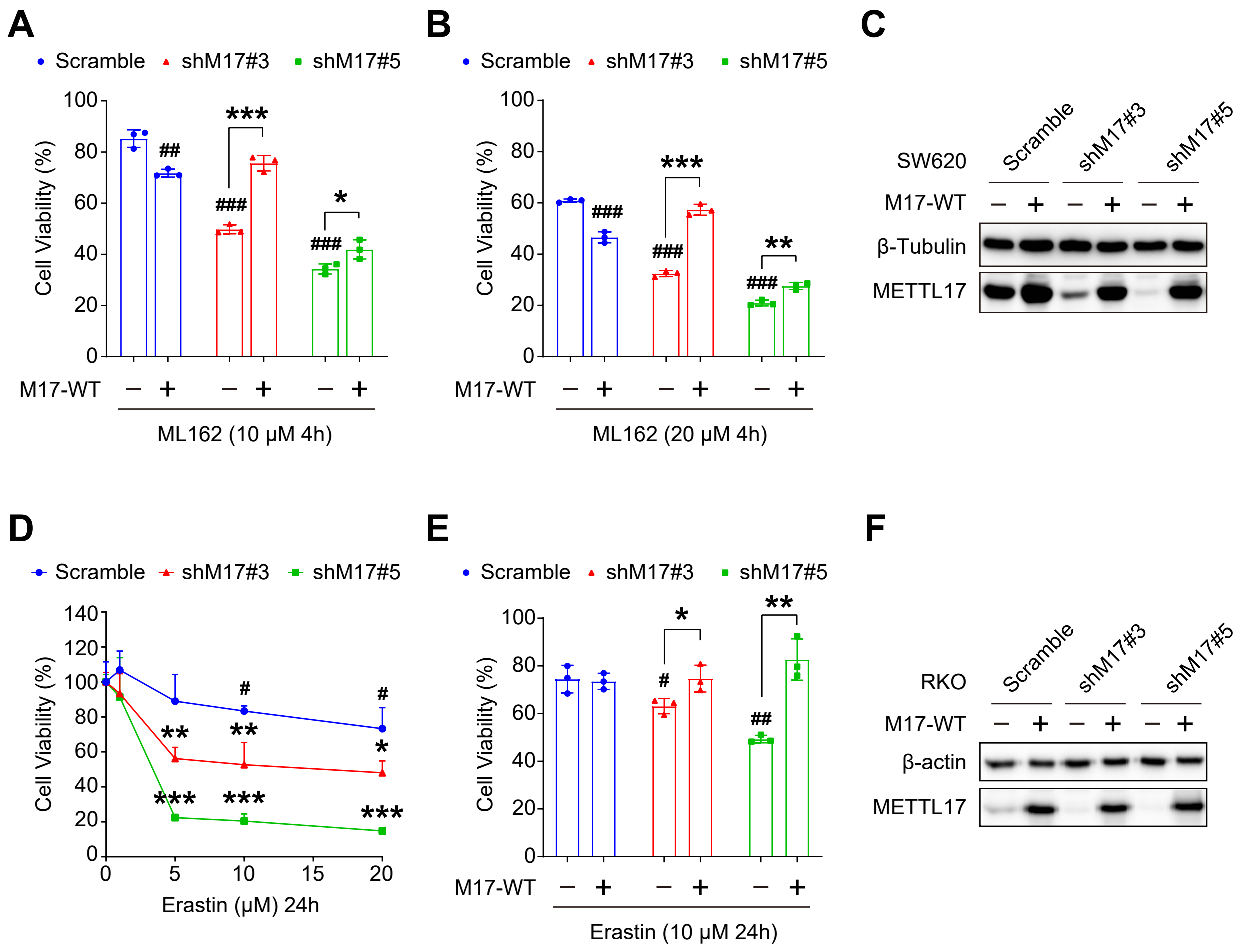

Supplementary Fig.3 Knockdown of METTL17 inhibits migration, invasion, and colony formation of CRC cells.

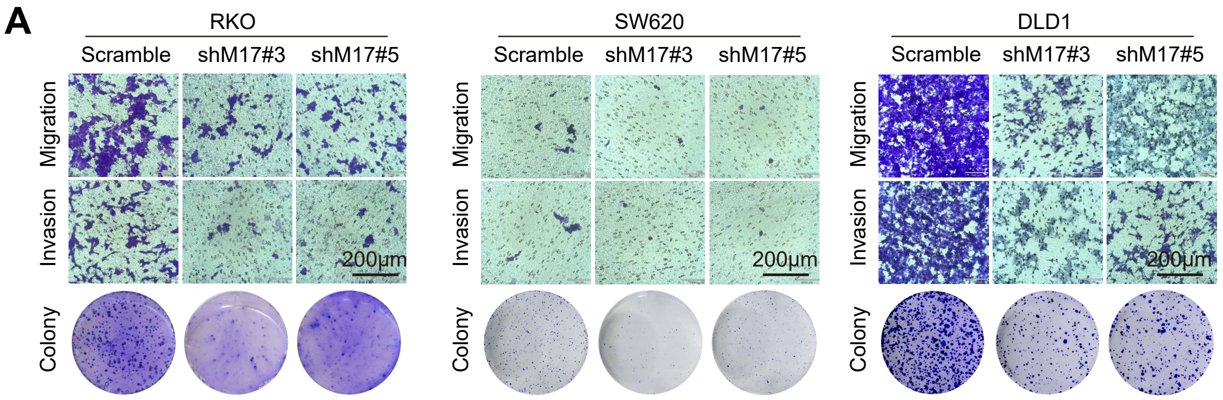

Supplementary Fig.4 Knockdown of METTL17 inhibits oncogenic pathways.

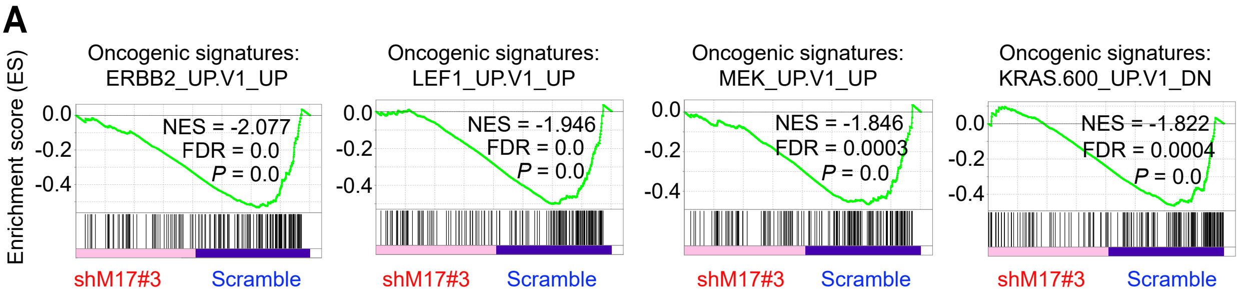

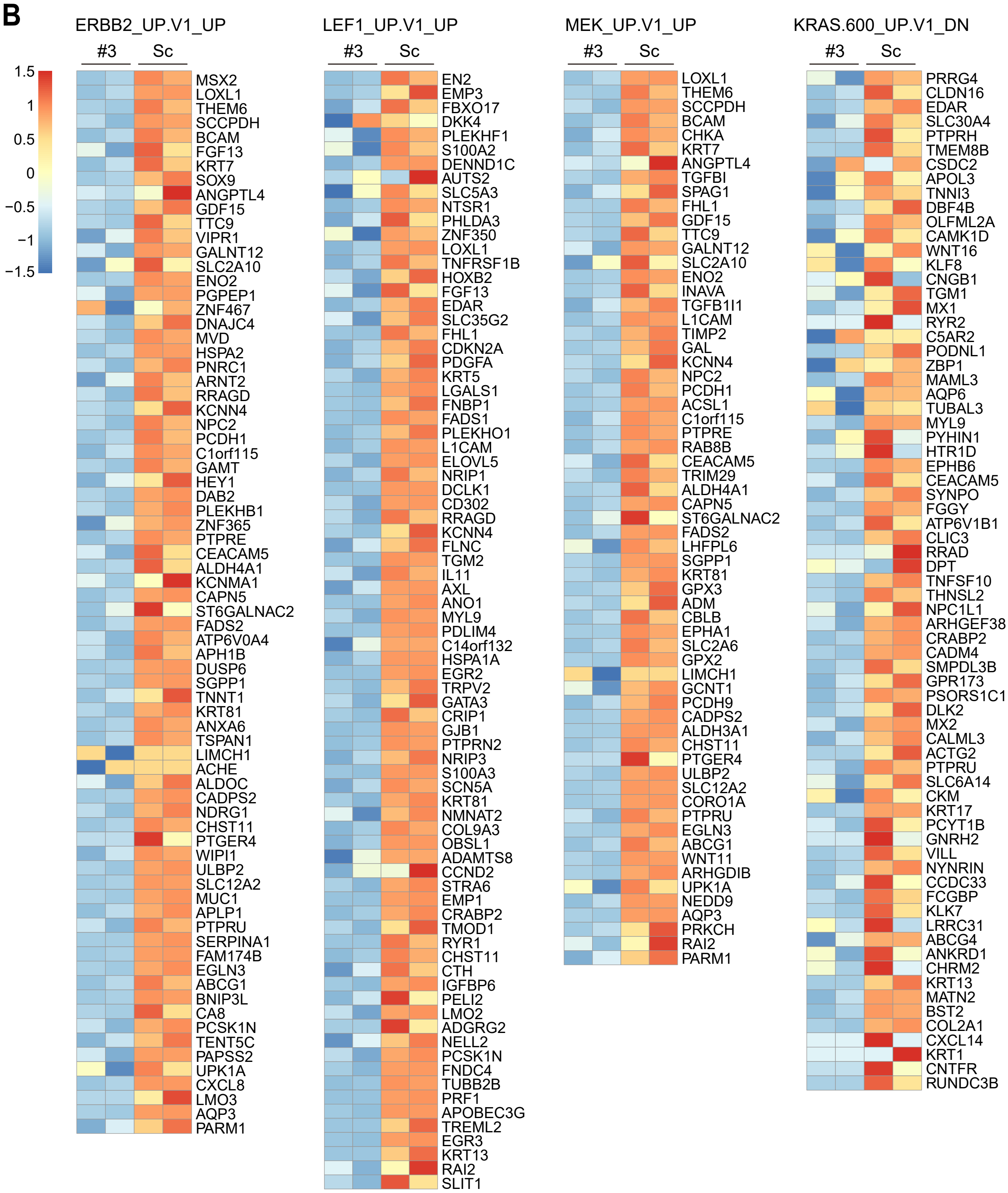

Supplementary Fig.5 *Mettl17*$^{-/-}$ mice are nonviable, while *Mettl17*$^{+/-}$ mice exhibit normal colon development.

A

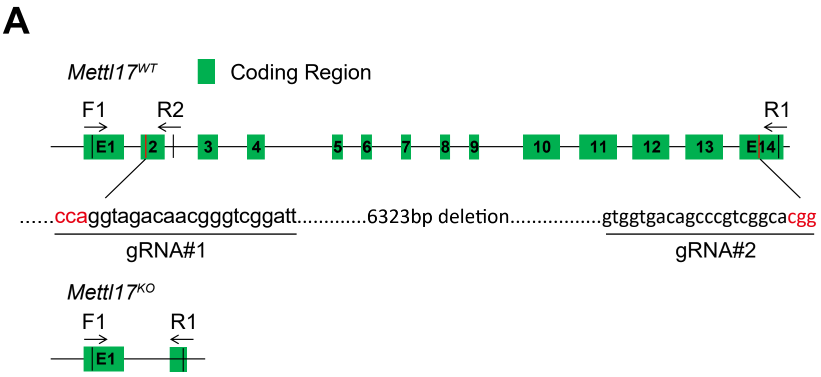

B

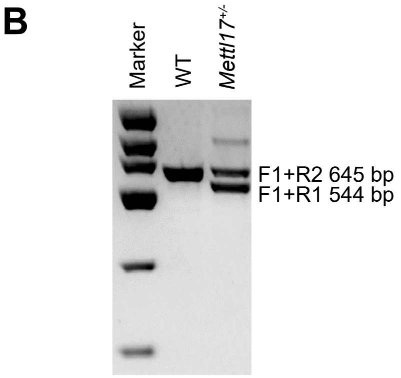

C

Age of Mice	*Mettl17*$^{-/-}$	*Mettl17*$^{+/-}$	WT	Total number
Embryo day 12.5	0	22	10	32
Postnatal day 7	0	65	31	96

D

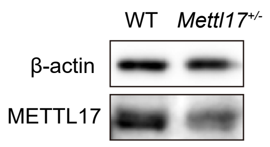

E

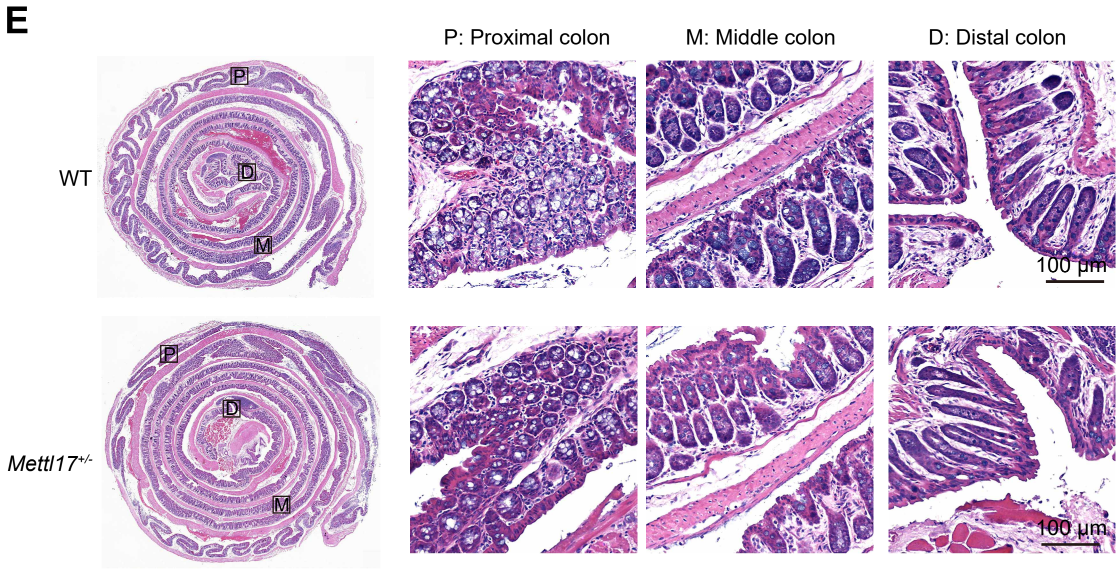

Supplementary Fig.6 METTL17 knockdown promotes lipid peroxidation and ROS formation in both the cytoplasm and mitochondria.

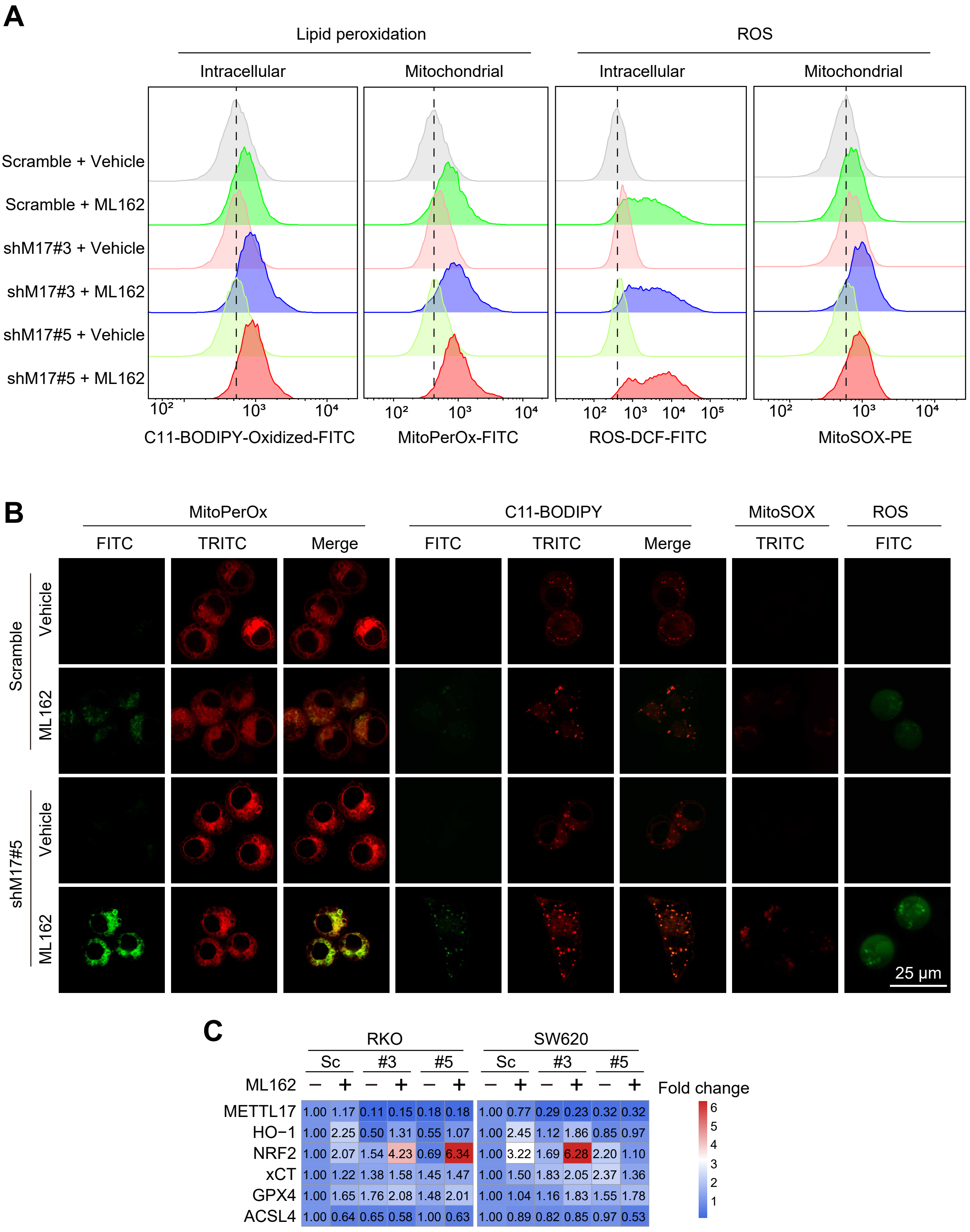

Supplementary Fig.7 Knockdown of METTL17 doesn't affect RNA levels of mitochondrial mRNA.

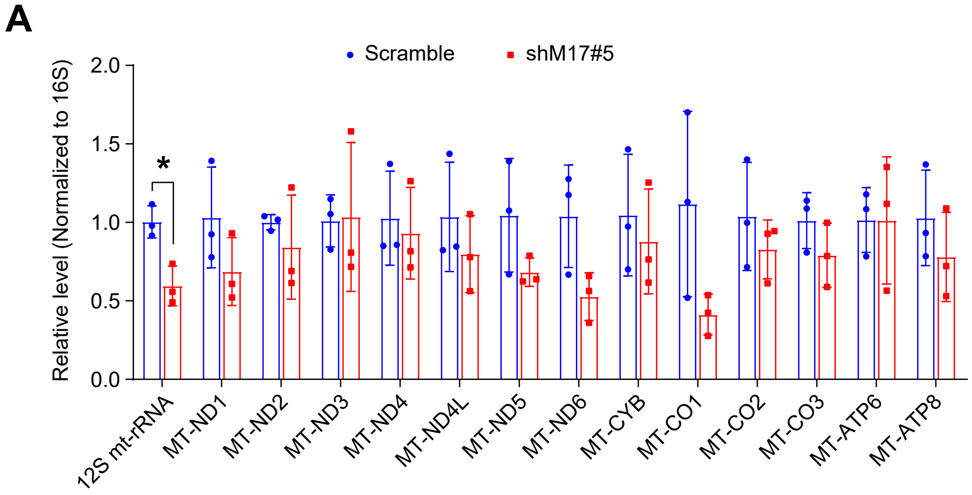

Supplementary Fig.8 Knockdown of METTL17 doesn't affect the expression of mitochondrial RNA methyltransferase.

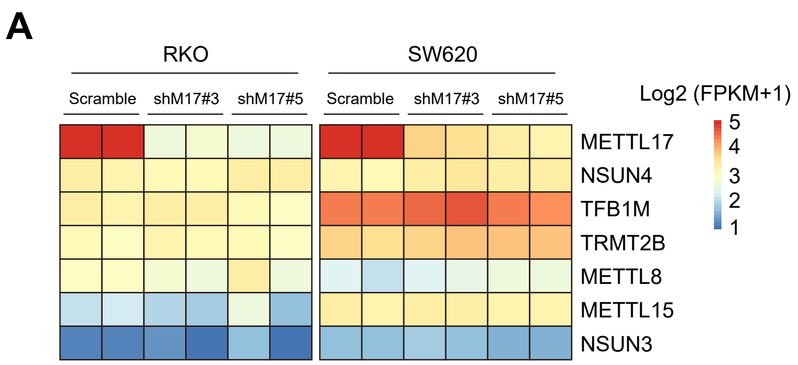

Supplementary Fig.9 METTL17 interacting proteins are essential for mitochondrial gene expression and knockdown of METTL17 doesn't affect their expression in CRC cell lines.

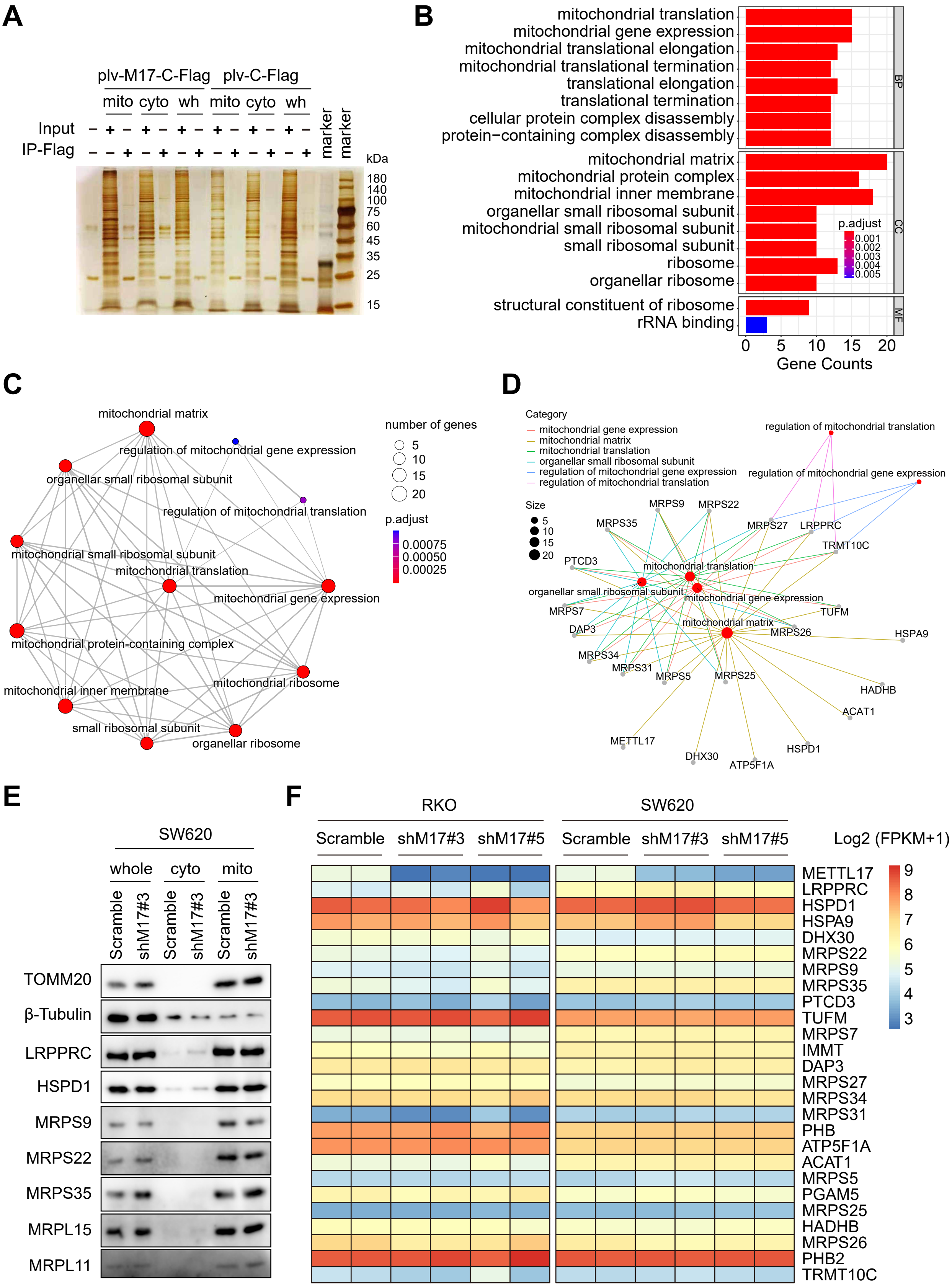

Supplementary Fig.10 The expression of LRPPRC, MRPS9, MRPS22, and MRPS35 positively correspond with cancer cell resistance to ferroptosis.

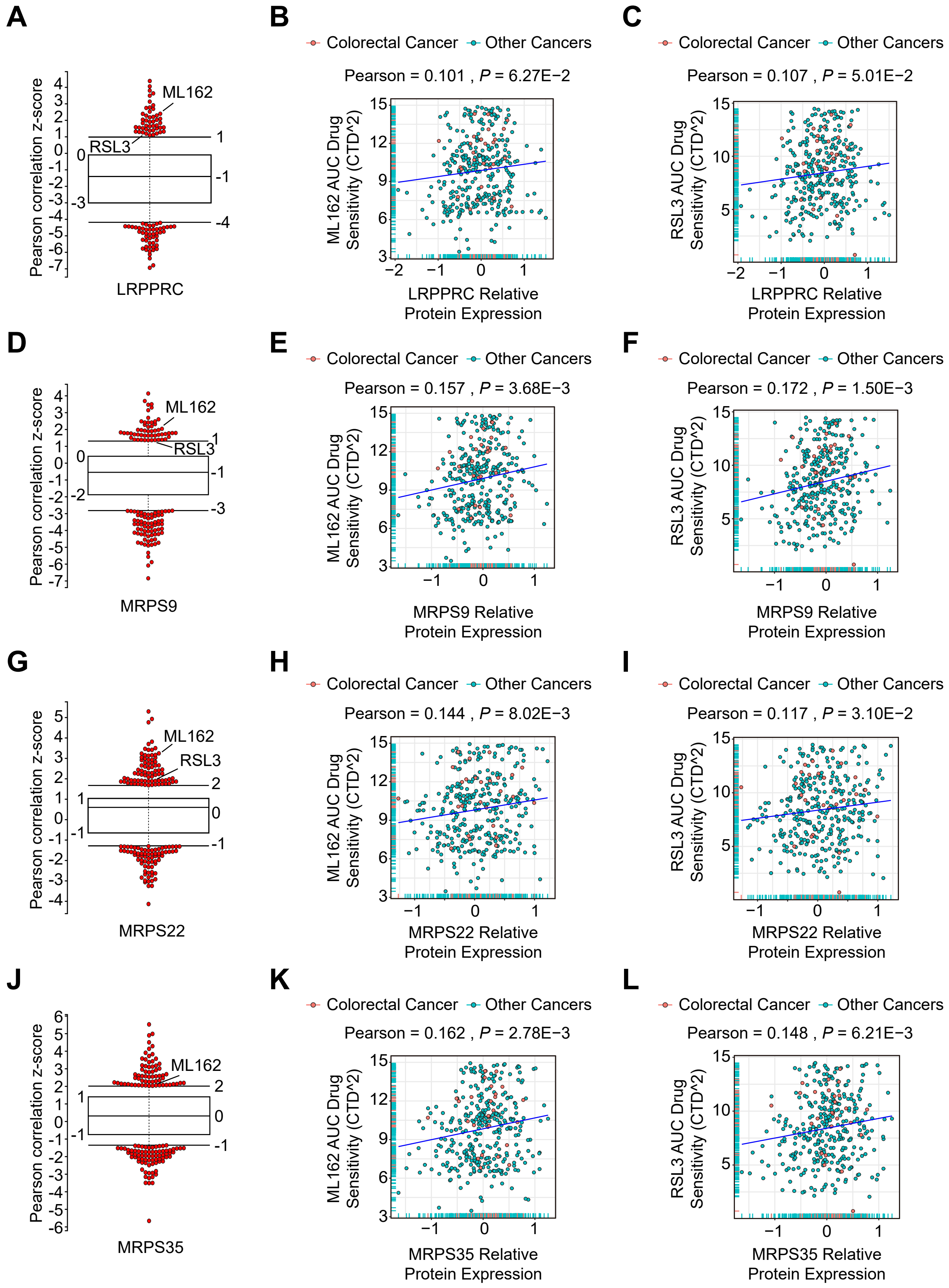

Supplementary Fig.11 DepMap analysis shows a high correlation between METTL17 and its interacting proteins in dependency gene effects of cancer cells.

A

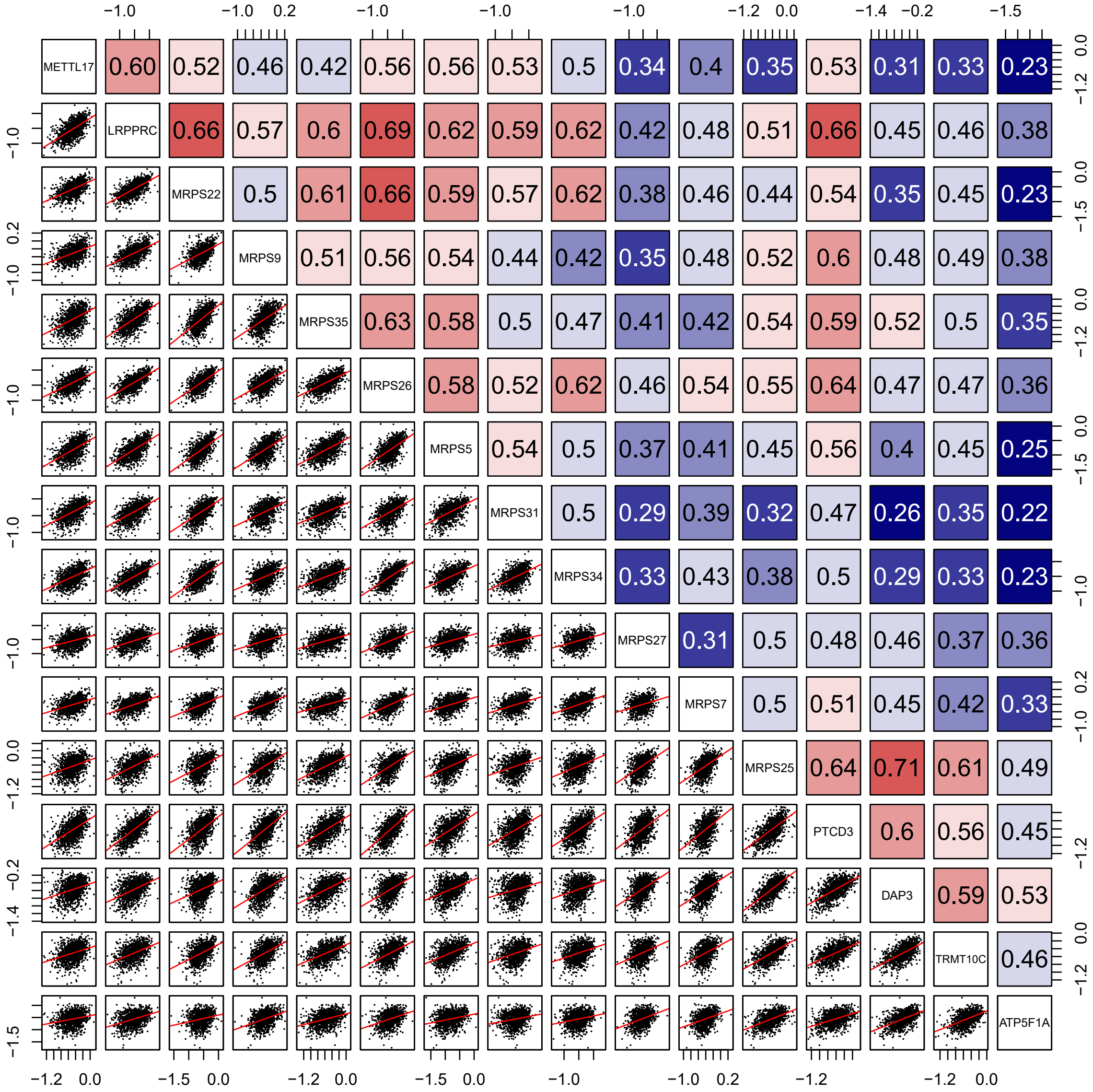

Supplementary Fig.12 The relationship between the expression and gene effect of METTL17 in CRC cell lines.

A

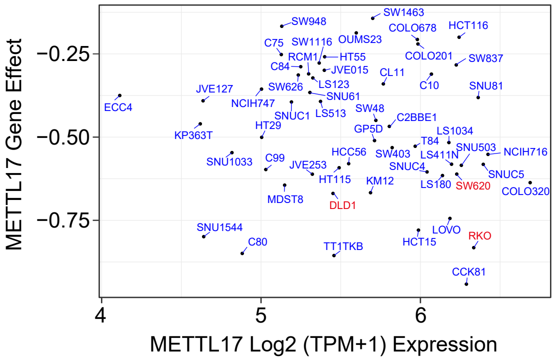

Supplementary Fig.13 METTL17 loss inhibits the gene expression related to oxidative phosphorylation.

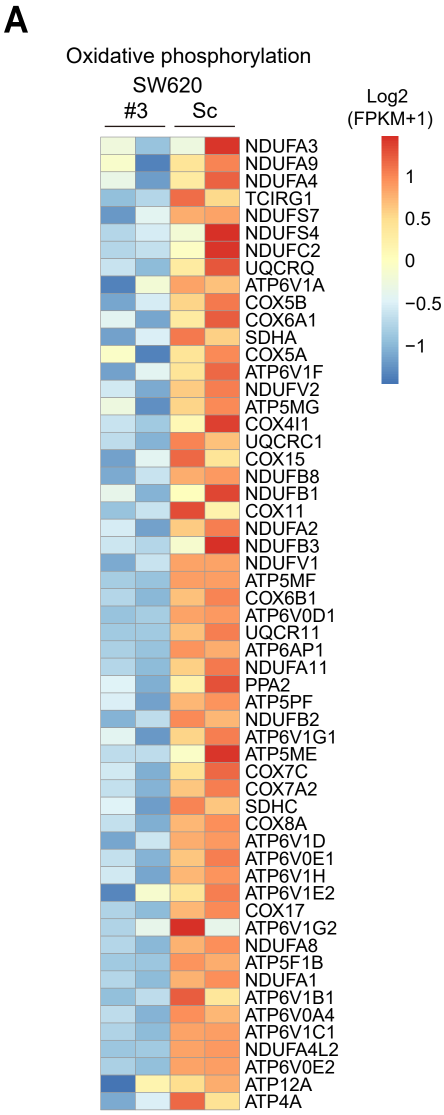

Supplementary Fig.14 The expression profiling of genes related to cell redox homeostasis.

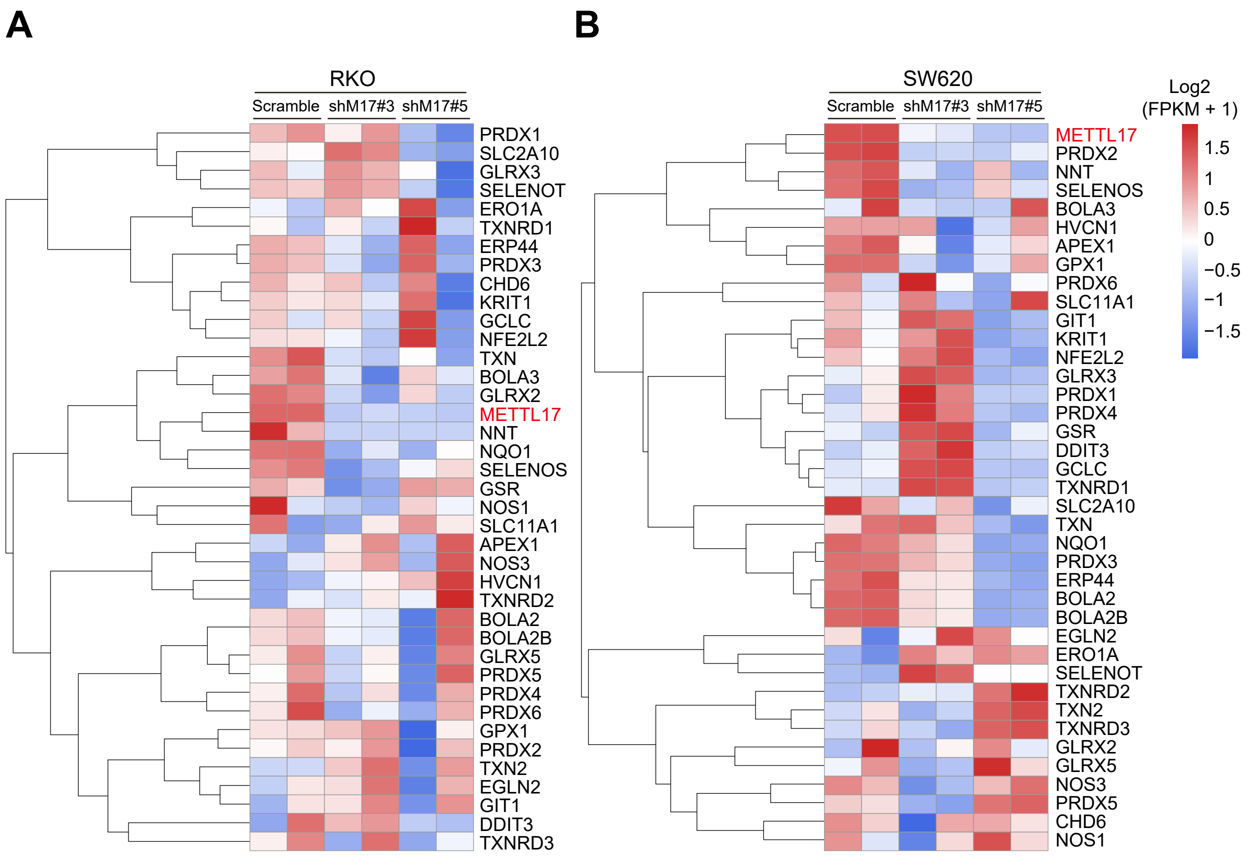

Supplementary Fig.15 The METTL17 co-dependent genes enrich in the mitochondrial related pathways.

A

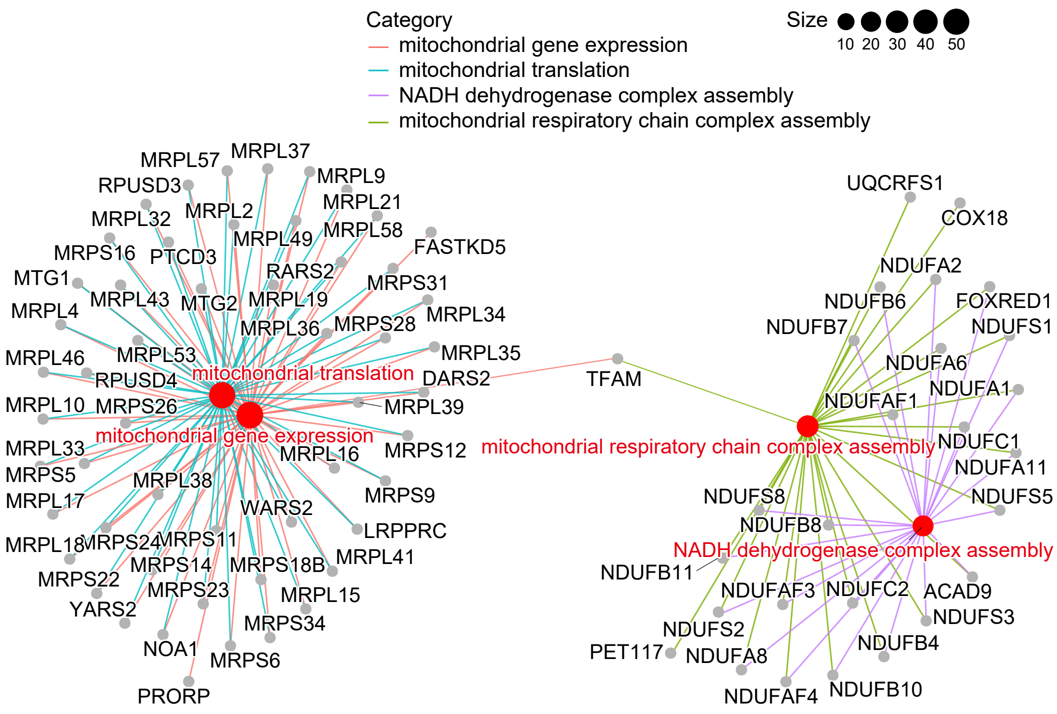

Supplement: Multimedia component 1 [file mmc1.pdf]
